# Generalization, Cross-ICU Transfer, and Explainability of a Mortality and Time-to-Discharge Framework for the Intensive Care Unit

**DOI:** 10.3390/jcm15186957

**Published:** 2026-09-08

**Authors:** Àlex Pardo, Josep Gómez, Julen Berrueta, Alejandro García-Martínez, Pau Orts, Sara Manrique, Alejandro Rodríguez, María Bodí

**Affiliations:** 1 Department of Medicine and Surgery, Rovira Virgili University, 43201 Tarragona, Spain; juberrueta.hj23.ics@gencat.cat (J.B.); ports.hj23.ics@gencat.cat (P.O.); smanriquemoreno@gmail.com (S.M.); ahr1161@yahoo.es (A.R.); mbodi.hj23.ics@gencat.cat (M.B.); 2 Critical Care Department, Joan XXIII University Hospital, 43005 Tarragona, Spain; alejgarcia.hj23.ics@gencat.cat; 3 Pere Virgili Health Research Institute, Joan XXIII University Hospital, Rovira Virgili University, 43005 Tarragona, Spain

**Keywords:** intensive care, machine learning, external validation, transfer learning, generalizability, explainable AI, mortality, length of stay, critical care management

## Abstract

**Background:** PADS combines two neural networks predicting ICU mortality and discharge within 48 h, placing critically ill patients into one of four clinically meaningful states. Developed on MIMIC-IV alone, it left open whether it generalizes to other ICUs, whether its models transfer across hospitals, and whether its predictions can be explained at the bedside. **Methods:** We evaluated PADS on four ICU databases from different hospitals and countries (MIMIC-IV, AmsterdamUMCdb, eICU-CRD, and HiRID), using the same routinely collected variables. Mortality is scored on the final 48-h window (terminal-window, not early-warning, discrimination). For each external database, we compared the MIMIC model used as-is, retrained from scratch, and retrained from the MIMIC weights, and added an explainability layer. **Results:** For mortality, reusing and retraining the MIMIC model gave the highest discrimination on every database (AUROC 0.955–0.986; terminal-window (near-outcome) discrimination) and stabilized training; used as-is, it ranged from chance (Amsterdam) to good (eICU, HiRID). For discharge, training fresh on local data matched or beat reusing MIMIC on every external database, consistent with discharge timing depending on local organization rather than physiology. The explainability layer produced clinically coherent, cross-checked explanations. **Conclusions:** Transportability was task-dependent: mortality transferred between hospitals, discharge did not. PADS demonstrated promising external transportability across heterogeneous ICU databases, particularly after local adaptation. Reusing and adapting the MIMIC-IV mortality model across hospitals improves accuracy. This approach also stabilizes training, providing a basis for potential federated deployment, whereas discharge is better trained locally. The mortality results reported here are terminal-window discrimination and do not support use of the framework as an early-warning model. A transparent explainability layer provides an interpretable representation of model predictions, addressing a key barrier to clinical adoption.

## 1. Background

Moving a machine-learning prognostic model from the cohort where it was developed into the daily routine of an ICU is usually blocked by the same three questions: does the model still work in a different unit, can it be moved between institutions without sharing patient data, and can a clinician understand why a given prediction was made? In a previous study we introduced the Patient Outcome Assessment and Decision Support (PADS) framework [1], a pair of neural networks of a type well suited to data that change over time (i.e., LSTM) that, from a small set of variables collected during the daily care routine, predict ICU mortality and the probability of discharge in the next 48 h and combine them into a four-state stratification (alive/discharged in 48 h, alive/not discharged, dead in 48 h, or dead later) presented to clinicians as a continuous, hourly panel. PADS was deliberately built on a constrained and reproducible feature set (the SOFA variables plus age, admission type, and comorbidities) so that it could be implemented in any unit with an electronic health record (EHR). However, it was developed and evaluated using only one database, MIMIC-IV. The present work studies the generalization, transferability, and explainability of that framework.

Most ICU prediction models are reported on a single cohort, and their performance tends to drop when they are applied to a population they were not trained on, due to differences in case mix, measurement practice, unit policy, and EHR coding [2]. A well-known example is a widely deployed sepsis-prediction model that performed well in its development hospital but considerably worse when validated elsewhere [3]. Robust external validation, where a frozen model is evaluated on independent databases, is therefore a prerequisite for clinical use, but it is still relatively rare [4]. Among the works that attempt it, ref. [5] validated a short-term mortality model on MIMIC-III, eICU, and AmsterdamUMCdb, but did not release reproducible code or describe a bedside presentation. The growing availability of large and structurally different ICU databases that are freely accessible, such as MIMIC-IV (Boston, USA) [6], AmsterdamUMCdb (Amsterdam, The Netherlands) [7], the multicenter eICU Collaborative Research Database (USA) [8], and HiRID (Bern, Switzerland) [9], now makes a real multi-database evaluation possible.

When a model does not work well as it is, there are two options: train a new model from scratch on the local data, or reuse the model already trained on the source database and adapt it locally (transfer learning). Transfer learning is particularly useful in critical care for three reasons: the relationship between a patient’s physiological condition and their outcome is, in principle, similar across ICUs and may be expected to show some degree of generalizability [10]; the target ICU may have far fewer patients than the development cohort, so starting from an existing model helps; and sharing a trained model instead of patient records fits well with federated arrangements between hospitals [11,12], where data never leave each institution. How much of the model should be adapted locally, and whether transfer helps every outcome to the same extent, are questions that have not been systematically answered for a combined mortality and discharge framework.

The third open question concerns interpretability. Neural network models for the ICU are frequently criticized as opaque, and the lack of a clear and trustworthy explanation is a recognized barrier to clinician trust and to regulatory acceptance [13,14]. In the first PADS study, we reported that explainability tools had been tested without success. Explaining this kind of model is not trivial, because a prediction depends at the same time on which variable mattered and on when during the stay it happened, so a simple list of “important variables” is not enough. In addition, an explanation can easily look convincing while actually being wrong, so the method used has to be checked carefully (Section 2.6).

The objective of this work is therefore threefold: first, to externally validate the PADS mortality and discharge models on four heterogeneous ICU databases; second, to determine, for each task, whether and how the MIMIC-trained weights transfer, comparing direct transfer, training from scratch on local data, and three fine-tuning strategies; and third, to add a transparent explainability layer with internal consistency checks that produces per-patient and cohort-level explanations suitable for the bedside.

## 2. Methods

### 2.1. Data Sources

We used four publicly accessible, de-identified ICU databases. MIMIC-IV [6] (Beth Israel Deaconess Medical Center, Boston) is the database used to develop the original PADS models, and here it serves both as the source database (the one the original models were trained on) and as an internal reference. AmsterdamUMCdb [7] (Amsterdam UMC, The Netherlands) is a comprehensive ICU database. The eICU Collaborative Research Database (eICU-CRD) [8] gathers stays from many US hospitals, so it allows us to test heterogeneity within a single country across multiple centers. HiRID [9,15] (Bern University Hospital, Switzerland) is a high-resolution database, and we treat it as the most demanding external target because it does not natively contain all of the framework’s input variables (see Section 2.3). All four are accessed through PhysioNet [16] or the corresponding data-use agreement.

Following the inclusion criteria of the original framework, we kept stays of at least 48 h (the minimum window the models require) and excluded those with life-support treatment-limitation orders where this information was available. Each database was split into training and held-out test stays (around 80/20), at random by stay rather than grouped by hospital. Unlike the first study, which used 5-fold cross-validation on a single database, here we used a single held-out test set per database, because the study already spans four databases and several retraining strategies. To make sure the results were not due to a particular split or starting point, the models trained from scratch were retrained with several random seeds (Section 2.5). The resulting cohorts are summarized in Table 1.

### 2.2. Framework and Base Models

We reuse, without any change, the two PADS LSTM networks and their architecture from the first study [1]: an LSTM layer (100 units), two dense layers (20 and 10 neurons with ReLU activation), and a two-class softmax output, with one network predicting ICU mortality and the other discharge in the next 48 h. The mortality network is applied to the last 48 h of a stay, and the discharge network is applied at several anchor points. In deployment, both networks are re-evaluated every hour, which is the frequency at which the underlying variables are recorded, so the four-state panel is refreshed hourly for as long as the patient stays in the unit. Throughout this paper, the base model is the MIMIC-trained pair shipped with the framework, together with the MIMIC normalizers. The combined four-state visualization and the custom error-scoring scheme are described in [1]; this paper focuses on the discrimination, transferability, and explanation of the two component models, but also reports the combined four-state error on each database as a first check of the integrated product (Section 3.6).

### 2.3. Feature Harmonization and ETL

Each database was preprocessed to obtain the 16 variables needed by the framework: 11 time-varying SOFA-related variables (the two PaO_2_/FiO_2_ ratios for the ventilated and non-ventilated states, the Glasgow Coma Score, the four vasopressor rates, the minimum mean blood pressure, the maximum bilirubin, the minimum platelets, and the maximum creatinine) plus age, admission type, and the Charlson comorbidity index. Missing-value imputation followed the original protocol [1]. Outliers in the 11 time-varying variables were clipped to fixed, clinically defined physiological bounds (e.g., mean blood pressure 10–150 mmHg, creatinine 0.2–20 mg/dL, platelets 5–2000 × 10^9^/L) taken unchanged from the original protocol [1] and applied identically across all four databases, rather than to percentiles computed on each database’s own data. Per-feature scaling, by contrast, was recomputed on each database’s own data for the four retrained conditions, while the direct-transfer condition kept the original MIMIC-IV scaling (see Section 2.4). Missing values in the 11 time-varying variables were handled by one of two fixed, per-variable rules. The two PaO_2_/FiO_2_ fields and the four vasopressor rates were filled with 0, since the absence of an active order or measurement is clinically equivalent to a rate or ratio of zero for these fields. The Glasgow Coma Score, mean blood pressure, bilirubin, platelets, and creatinine were instead carried forward from the previous hour of the same stay (last-observation-carried-forward, LOCF). Because LOCF cannot fill a stay’s very first hour, that hour was pre-filled for these five variables using each variable’s global median, computed once across all stays’ first 48 h in the training data and reused unchanged at inference time. Age, the Charlson comorbidity index, and admission type are static per-stay fields with no imputation rule; a database lacking one of these fields entirely used a single constant value for every stay instead (see below and Appendix A). The pipeline does not interpolate between hours and does not add any missingness indicator. How much remains to be filled after these rules varies considerably from one database and one variable to another; the detail per variable and per database is given in Appendix B (Table A1).

Not every variable was available in every database. eICU-CRD provided all 16. In AmsterdamUMCdb, the Charlson comorbidity index was not available and was set to a constant value, so in practice 15 of the 16 variables carried information. HiRID was the most incomplete: 5 of the 16 inputs were not available (the dopamine rate, the Charlson index, and the three admission-type categories) and were set to constant values, using the dopamine rate at 0 (which is harmless, since dopamine is 0 in 99.4% of the MIMIC records), the Charlson index at the average value in MIMIC (5.78), and the admission type as “Medical” (the most common value in MIMIC, with 87% of the records). Importantly, retraining lets the model re-learn from the local data and ignore any variable that is always constant, so these missing variables only affect the results when the original MIMIC model is used without retraining; we flag this wherever it is relevant. The ETL adapters are released together with the code, and a per-variable mapping of each input to its source field and unit in every database is given in Appendix A.

### 2.4. Transfer-Learning Conditions

Transfer learning, in which a model trained on one dataset is reused as the starting point for another rather than training a new one from zero, is a well-established technique outside medicine. It is standard practice in computer vision, where networks pre-trained on large image collections are fine-tuned on smaller target tasks [17,18], and in natural language processing, where large pre-trained models are adapted to more specific downstream tasks [19]. For each external database, we evaluated five conditions:

▪ Direct transfer (zero-shot): the original MIMIC model applied directly to the new database, without any local retraining. This measures how well it transfers as it is.
▪ Scratch: a new model trained from scratch on the target database. This measures what the local data alone can achieve.
▪ Full: start from the MIMIC model and retrain all of it on the target database.
▪ Dense: start from the MIMIC model, keep the part that reads the time patterns (the LSTM layer) fixed, and retrain only the final layers that turn those patterns into a probability.
▪ LSTM: start from the MIMIC model, keep the final layers fixed, and retrain only the LSTM layer.

For the four retrained conditions, the scaling of the variables was recomputed on each database’s own data, while the direct-transfer condition keeps the original MIMIC scaling. Comparing the last three conditions tells us which part of the model needs to change for a new hospital: the part that reads the time patterns of the physiological variables (the LSTM), or the part that converts them into a final probability (the dense layers). All retrained mortality models, whether trained from scratch or fine-tuned from the MIMIC weights, used the same imbalance handling—a class-balanced focal loss together with balanced class weights—so the conditions differ only in their starting weights, not in how class imbalance was treated.

### 2.5. Evaluation Protocol

We measured how well each model separates the patients who will have the outcome from those who will not, using the area under the ROC curve (AUROC) on a held-out test set that the model never saw during training. An AUROC of 1.0 indicates perfect discrimination, whereas an AUROC of 0.5 indicates performance no better than chance. Because the mortality network is applied to the final 48-h window of the stay, the mortality AUROC reported here reflects terminal-window (near-outcome) discrimination; it is not an early-warning performance and should not be read as one.

For each value, we also report a 95% confidence interval, which indicates how much the result would be expected to vary; it is obtained by repeatedly re-evaluating the model on random samples of the test patients (a procedure known as bootstrapping). The models trained from scratch turned out to be sensitive to the random starting point on the mortality task, so we retrained them several times with different random seeds and report the mean and standard deviation. The other conditions were stable and are reported once.

One caveat applies to the discharge confidence intervals. Each patient contributes several time windows to the discharge evaluation, and these windows are not independent of each other, so the discharge intervals come out narrower than they really are and should be read only as indicative. The discharge values themselves are not affected, and mortality (one value per patient) is not affected at all.

### 2.6. Prediction-Explainability Layer

**Figure 1:**
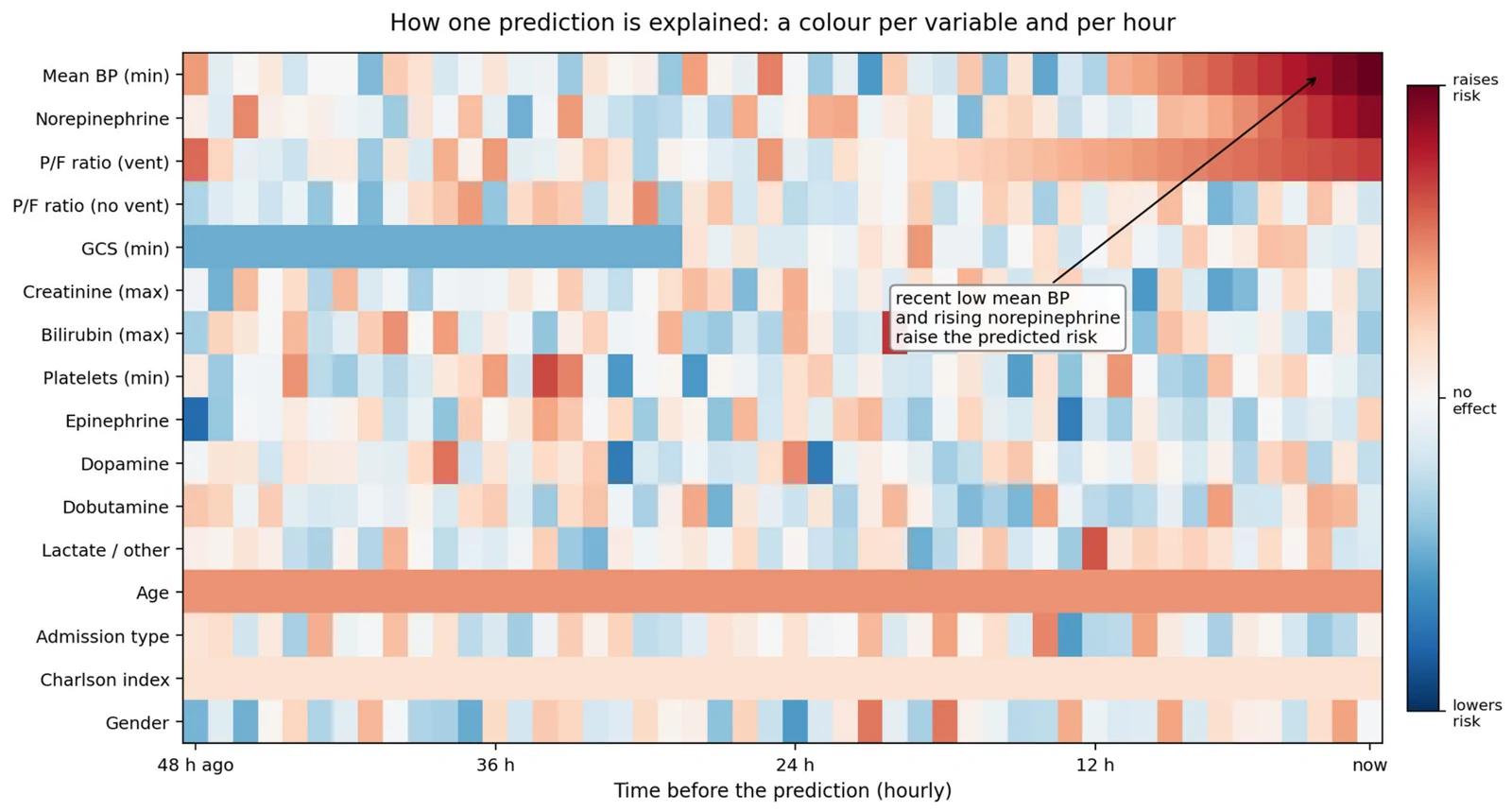
Illustration—not real data—of how a single prediction is explained. Each row is one of the 16 variables, and each column is one hour of the 48 h before the prediction. Red cells raised the predicted risk and blue cells lowered it. In this example, the recent drop in mean blood pressure together with rising norepinephrine (red, on the right) is what drives the high-risk prediction, while the good Glasgow Coma Score early in the stay (blue, on the left) lowered it.

We added an explainability layer that shows, for any single prediction, which variables pushed the risk up and which pushed it down, and at which hours of the 48-h window. The result is a colored map with one row per variable and one column per hour (Figure 1): red means that variable, at that hour, increased the predicted risk, blue means it decreased it, and white means it had little effect. The same map can be summarized as which variables mattered most overall (adding up across the hours) and as when the model was paying most attention (adding up across the variables). For variables that do not change during the stay (for example, age or admission type), the result is a single overall effect rather than an hour-by-hour pattern. The most useful view for the bedside places each important variable’s actual measured values, together with its normal range, next to this coloring, so the explanation can be read directly on the patient’s own trajectory.

The map is computed with three independent and well-established techniques, and we check that they agree, which provides evidence of internal consistency of the explanation. The first, Integrated Gradients [20], is the primary, state-of-the-art technique: starting from a typical patient, it gradually moves each variable towards the present patient’s actual value and adds up how much the predicted risk changes along the way, so that every variable receives a share of the risk that reflects how much it contributed. The second, Occlusion [21], is an intuitive check that hides one variable at a time (replacing it with its typical value) and measures how much the prediction moves: the more the risk changes when a variable is removed, the more the model was relying on it. The third, SHAP [22], is a secondary cross-check that comes from game theory and shares the risk fairly among the variables by asking, across many combinations of them, how much each one adds on average. When all three methods point in the same direction for the most influential variables, this provides evidence of internal consistency of the explanation.

Producing an explanation that is faithful to the model, and not merely plausible, requires some care. In particular, the comparison is always made against a realistic “typical patient” from the same ICU rather than against an empty record, because an empty record would correspond to a physiologically impossible patient (for example, a mean blood pressure of 0 mmHg and a GCS of 0) and would distort every explanation. Explanations are produced only for the models that perform well (the locally adapted ones) and for the most decision-relevant moment of the stay (the final window for mortality, and the last prediction before discharge).

Before any output is trusted, the layer runs a set of automatic checks. The most important one confirms that the explanation refers to exactly the model that produced the reported results, and not to a slightly different copy. Other checks confirm that the time direction is correct (so that “recent” really means the last hours), that the three techniques agree, and a clinical sanity check: a patient who died while on vasopressors with a low blood pressure must have those variables flagged as increasing the risk. Rules of this kind should be established together with clinical experts. All the checks passed for the four databases on both tasks.

### 2.7. Use of Generative Artificial Intelligence

During the preparation of this manuscript, the authors used Claude Opus 4.8, Gemini 2.5 Flash, and GPT-4o to make minor corrections to the text. The authors have reviewed and edited the output and take full responsibility for the content of this publication.

## 3. Results

The main result of this study is that the two tasks transfer in different ways: mortality transfers through the trained weights of the model, while discharge does not and is better learned locally. We present each task in turn, then the from-scratch stability result that supports the transfer argument, and finally the explainability layer. The full AUROC values with their intervals are in Table 2.

### 3.1. Direct-Transfer Generalization

**Figure 2:**
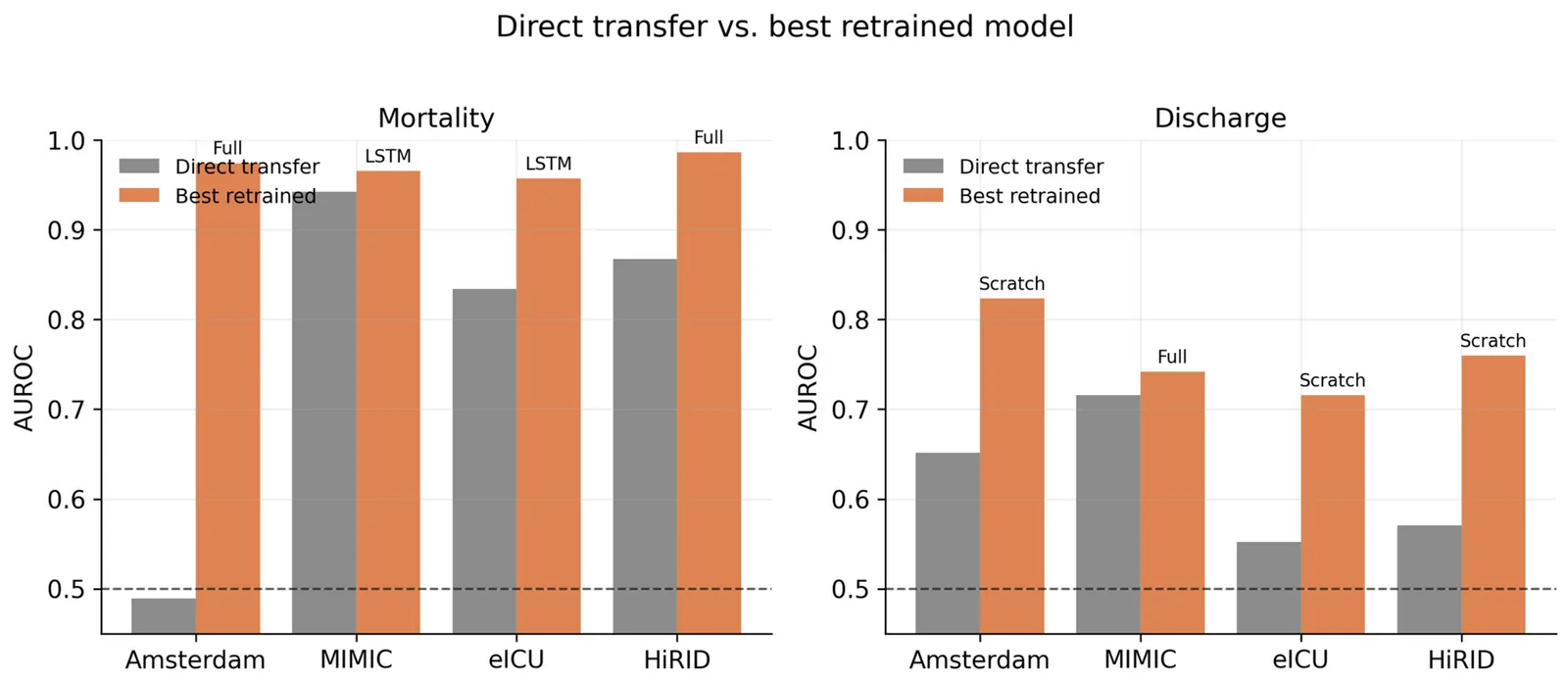
Direct transfer versus best-retrained AUROC for each database (mortality and discharge). Direct-transfer performance depends on the domain gap for mortality (performance approached chance level in Amsterdam) and is at or near chance for discharge on the external databases. Local adaptation recovers both.

Applied without any retraining, the MIMIC mortality model generalized to a degree that followed the distance between databases. It transferred well to the two databases closest to MIMIC in case mix and coding (that is, the type of patients admitted and how the measurements are recorded), eICU (0.834) and HiRID (0.868, despite the 5 imputed inputs), and it performed well on MIMIC itself (0.942), but it collapsed to chance on Amsterdam (0.489), the database that differs most from MIMIC in case mix and measurement practice. The per-variable harmonization (Appendix A) confirms that the inputs are mapped to the same clinical fields and units across databases (vasopressors in mcg/kg/min, mean blood pressure in mmHg, and so on), so this collapse reflects a genuine distribution shift, recoverable by local retraining, rather than a units or coding error. Direct-transfer discharge was uniformly weaker and at or near chance on the external databases (eICU 0.552, HiRID 0.570, Amsterdam 0.651), with only the MIMIC value, on its own database, being usable (0.716). Direct-transfer use is therefore not a safe deployment strategy: mortality is acceptable only where the domain gap is small, and discharge does not transfer at all without local adaptation (Figure 2). The within-database MIMIC figure should be read with care: the shipped MIMIC model was trained on the original MIMIC cohort, which overlaps the current test set (at least about 80% of these test stays, and all of them under the cross-validation protocol used originally), so the MIMIC direct-transfer value (0.942) can only be optimistic, which makes the gain from local retraining on MIMIC a conservative estimate; the external databases were never seen by the shipped model and are unaffected.

### 3.2. Mortality: The Trained Weights Transfer

**Figure 3:**
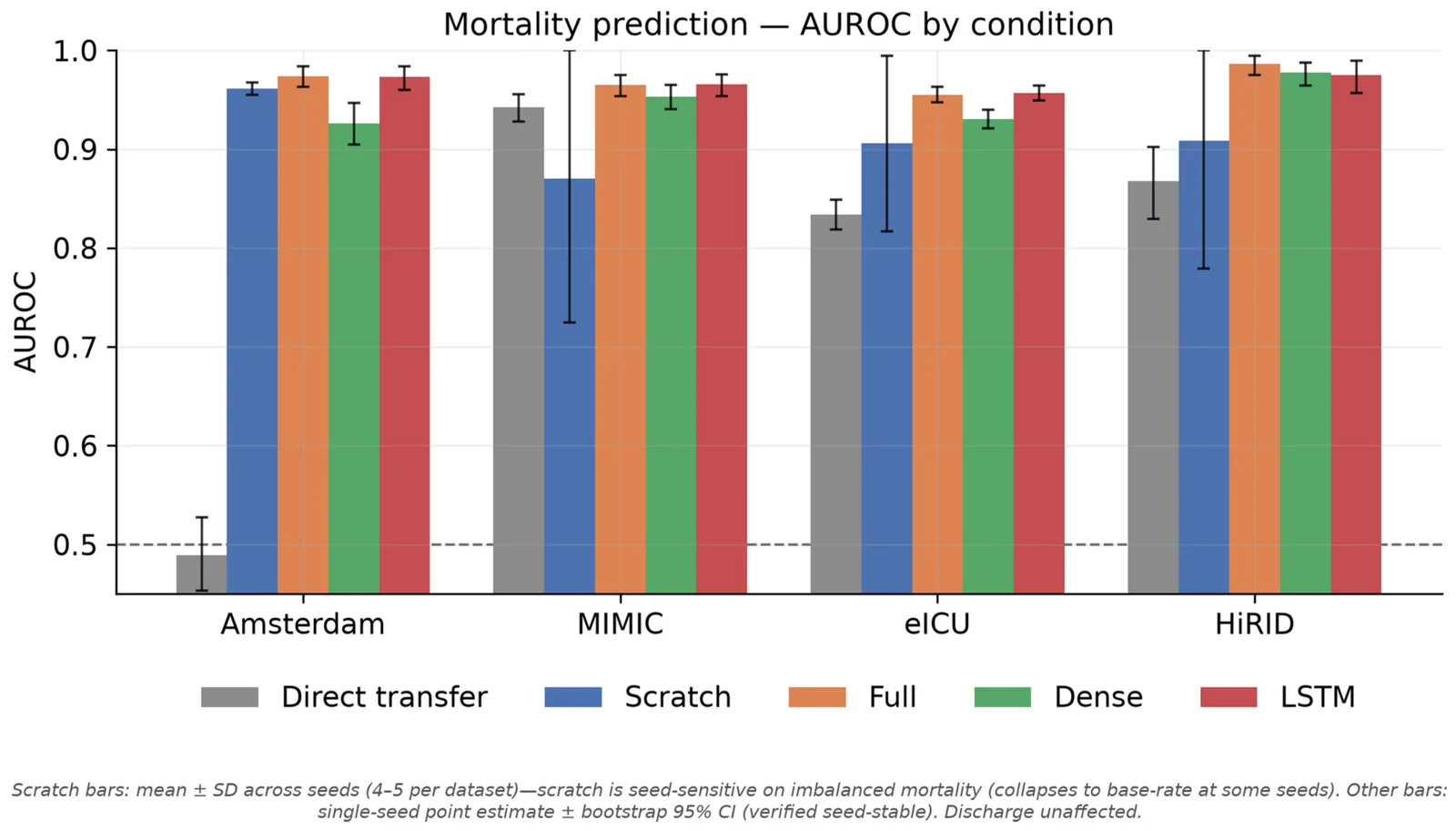
Mortality AUROC by condition for each database (error bars are the 95% bootstrap interval for single-seed cells, and the mean ± SD across seeds for scratch).

**Figure 4:**
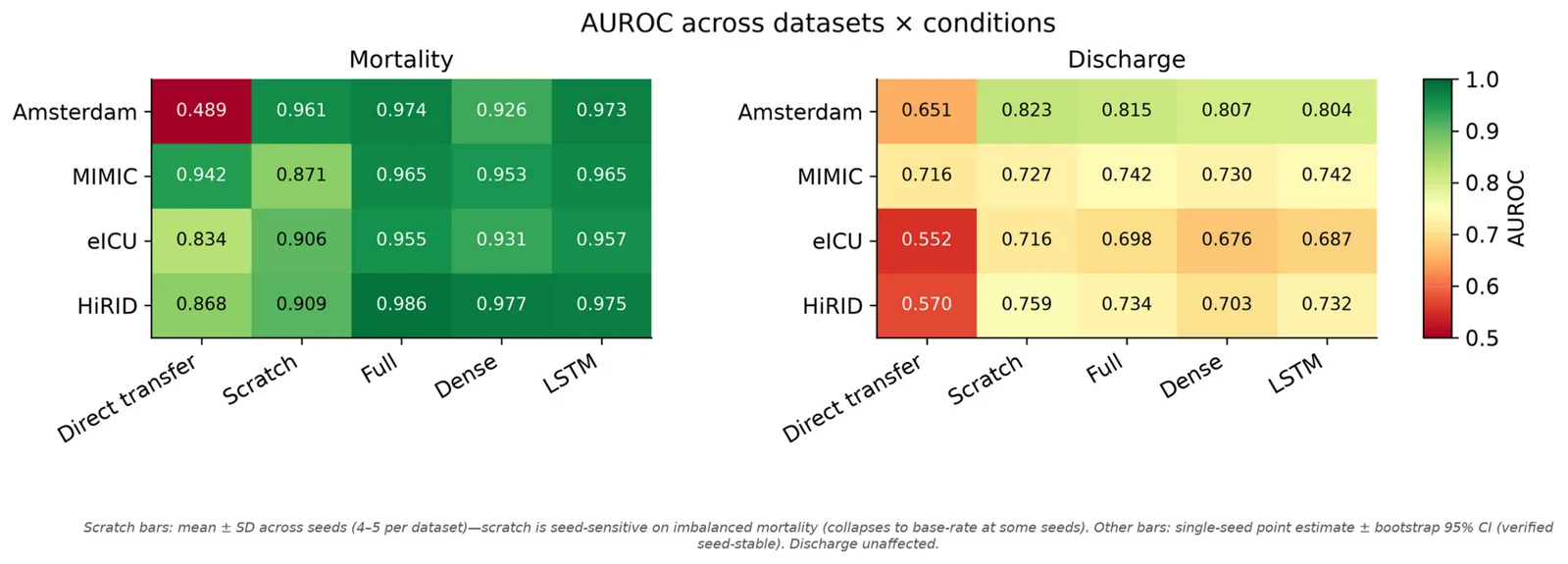
AUROC heatmap across all the databases and conditions (mortality and discharge).

**Figure 5:**
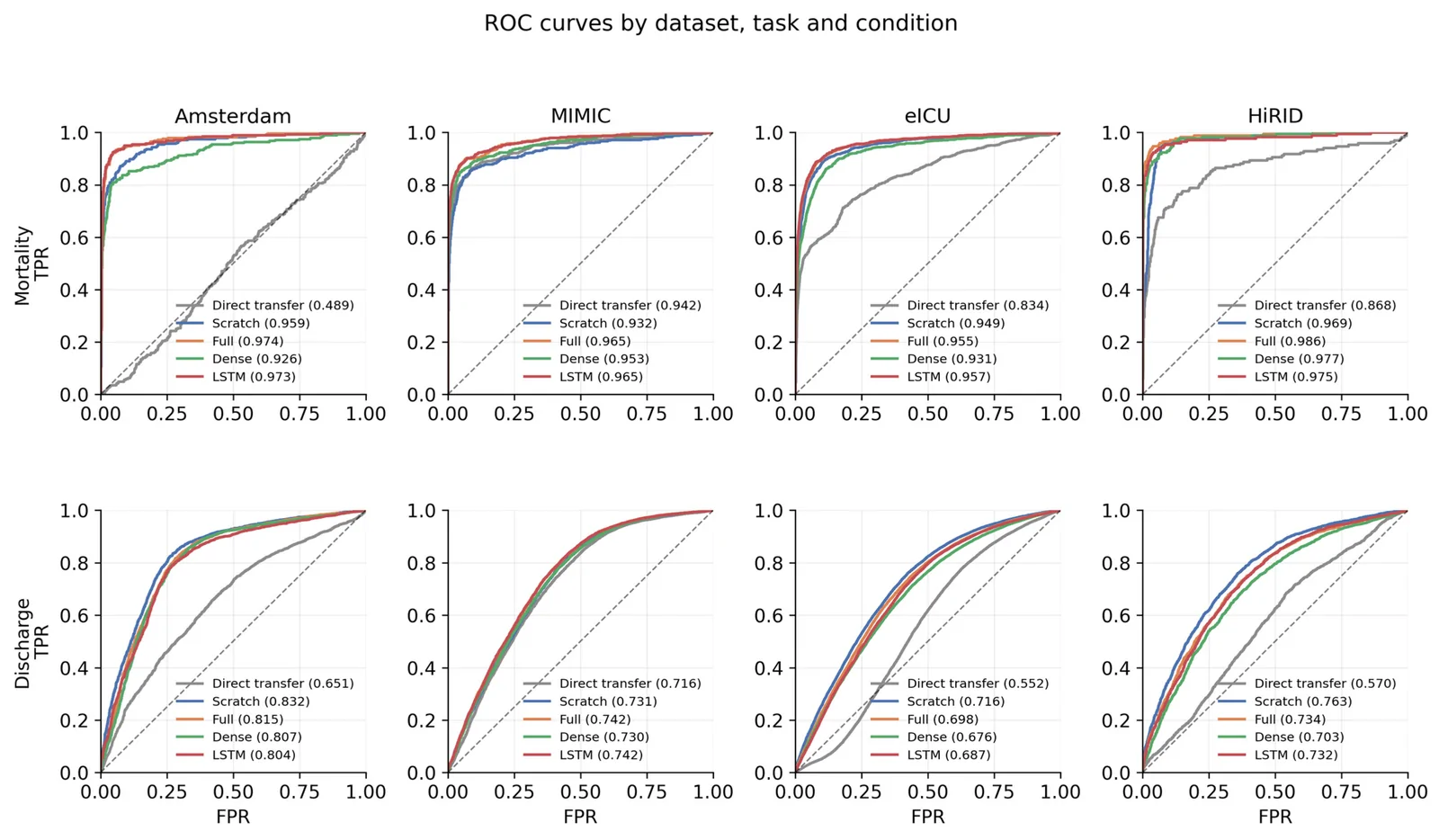
ROC curves per database and condition. Scratch is shown at the representative seed 42, while its bars in Figure 3 and Figure 6 report the across-seed mean ± SD, which is lower where one seed collapsed.

Retraining the MIMIC model on local data, starting from the MIMIC model rather than from scratch, yielded the highest or near-highest mortality discrimination across databases: retraining all of it reached 0.965 (MIMIC), 0.974 (Amsterdam), 0.955 (eICU), and 0.986 (HiRID, the highest mortality discrimination in the study; terminal-window (near-outcome) discrimination). That HiRID yields the highest mortality discrimination despite having 5 of its 16 inputs imputed to constants most likely reflects its case mix (a circulatory-failure-oriented cohort, per the companion study [15]) rather than any genuine model superiority, since local retraining learns from the variables that are present. The largest gain is on Amsterdam, where reusing the MIMIC model raises discrimination from 0.489 without retraining, which is close to chance, to 0.974, the highest value we observed on an external database. Retraining only the final layers and keeping the LSTM fixed (dense) was consistently a few points lower (for instance, 0.926 on Amsterdam and 0.931 on eICU), while retraining the LSTM was essentially as good as retraining the whole model (0.973 on Amsterdam, 0.957 on eICU). This tells us that the part of the model that reads the time patterns of the physiological variables (the LSTM) is what needs to be adapted to each hospital; adapting only the final layers is not enough. At the same time, starting from the MIMIC model rather than from scratch is what makes the result both accurate and stable (see Section 3.3). The pattern across conditions and databases is shown in Figure 3, Figure 4 and Figure 5.

**Figure 6:**
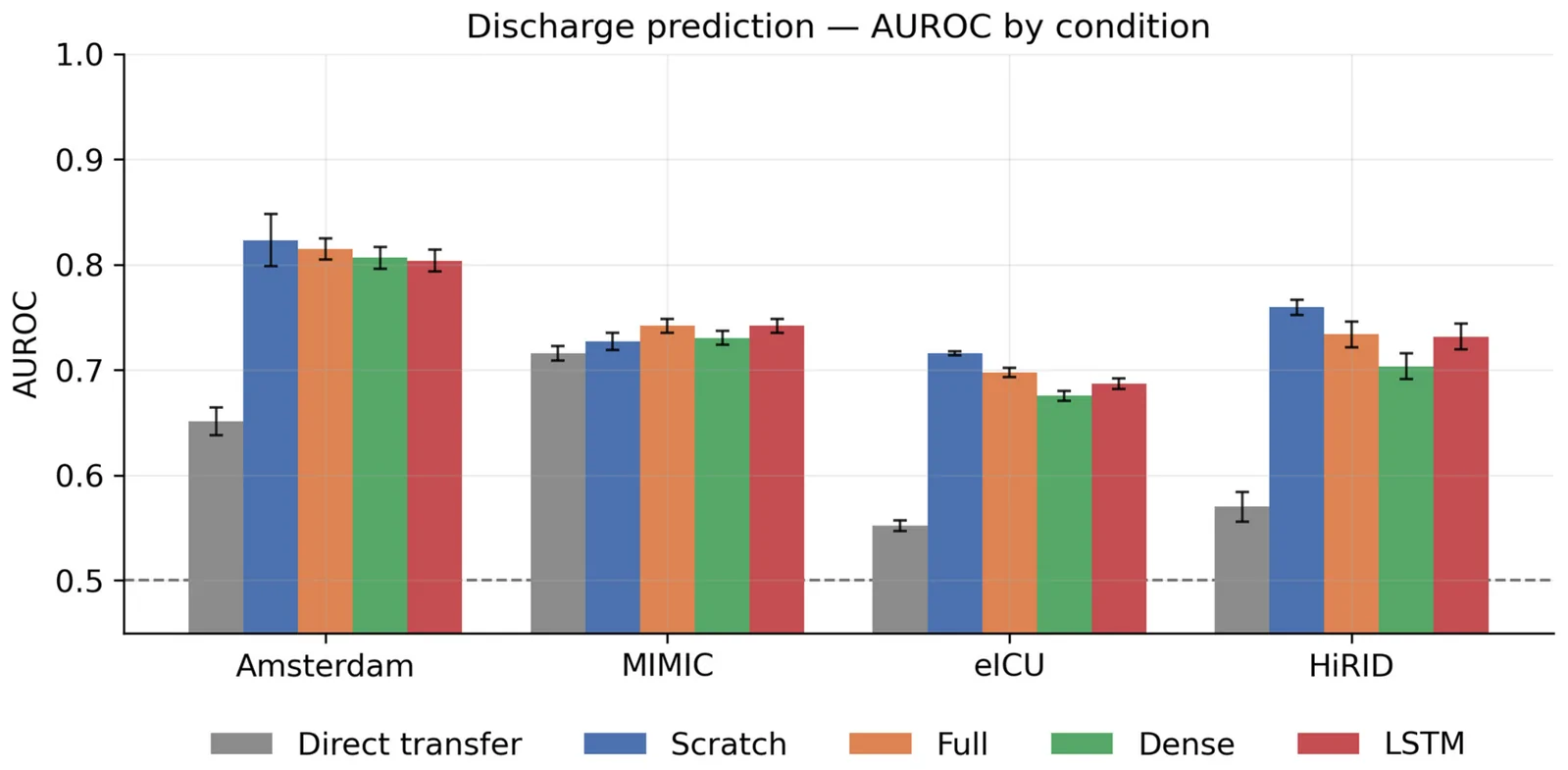
Discharge AUROC by condition for each database. On the three external databases, training from scratch on local data (seed-stable here) matches or exceeds fine-tuning the MIMIC weights.

### 3.3. Training from Scratch Is Unstable on Imbalanced Mortality

Training a new model from scratch worked well on average (mortality AUROC between 0.91 and 0.96), but performance was highly sensitive to the random initialization of model training. In some runs, the model failed to learn and simply predicted the average outcome for everyone. This happened on MIMIC (4 of 5 runs around 0.93, one run at 0.61), eICU (3 of 4 runs around 0.95, one run at 0.77), and HiRID (3 of 4 runs around 0.97, one run at 0.71). Amsterdam, where deaths are least rare (17% of patients), was the only database where this never happened (median 0.963 [0.953–0.967]). The problem is worse the rarer the death is in the data, and it disappears when training starts from the MIMIC model instead of from scratch. In other words, a shared pretrained initialization removes this residual instability, which persists even though both conditions used the same imbalance handling (a class-balanced focal loss with balanced class weights, Section 2.4); we did not test whether alternative initializations or resampling would remove it equally well, so this argues for transfer as a reliable fix under our already imbalance-aware protocol rather than as the only possible one. It also favors a federated arrangement in which hospitals start from a shared model and adapt it locally rather than each training on its own. Because a minority of seeds collapse, a mean ± SD is misleading here (it would give MIMIC 0.871 ± 0.146); Table 2 therefore reports scratch mortality as the median [range] over seeds—MIMIC 0.933 [0.610–0.939], with four of the five seeds around 0.93 and one at 0.61.

### 3.4. Discharge: The Architecture Generalizes but the Weights Do Not

For discharge, the picture is the opposite. On all three external databases, training a fresh model on local data matched or beat starting from the MIMIC model: 0.823 (from scratch) vs. 0.815 (starting from MIMIC) on Amsterdam, 0.716 vs. 0.698 on eICU, and 0.759 vs. 0.734 on HiRID. The from-scratch discharge models were also stable across runs. Only on MIMIC itself did starting from the MIMIC model do slightly better (0.742 vs. 0.727). Reassuringly, on MIMIC the discharge result reproduces the held-out test performance reported in the first paper (around 0.72; the original MIMIC model used here without retraining scores 0.716), confirming that the re-implemented pipeline reproduces the original model rather than changing it. That is, the PADS model learns discharge well in every ICU, but the MIMIC discharge model carries little that helps another hospital. This is expected, because the timing of discharge is driven by local organizational factors (bed availability, step-down capacity, unit policy, or national practice) rather than by the patient’s physiology alone. The practical recommendation therefore depends on the task: reuse and adapt the mortality model from MIMIC, but train the discharge model locally (Figure 6).

Because each stay contributes many correlated discharge windows, the row-level intervals in Table 2 overstate precision (Section 2.5); we therefore re-assessed the scratch-versus-fine-tuning difference with a bootstrap clustered by stay. On this de-correlated basis, the difference is small and not consistently in either direction: it marginally favors training from scratch on eICU (+0.005, 95% CI 0.002 to 0.008), shows no difference on MIMIC (+0.001, [−0.004, 0.005]), and marginally favors fine-tuning on Amsterdam (−0.017, [−0.025, −0.009]); HiRID could not be tested this way. We therefore do not claim a discrimination advantage for either training strategy on discharge. The recommendation to train discharge locally rests on the organizational nature of the outcome and on training from scratch being seed-stable and at least as good, not on a higher discharge AUC.

### 3.5. Explainability

The explainability layer passed all the automatic checks for the four databases on both tasks, including the most important one, which confirms that the explanations refer to exactly the models we evaluated and not to a slightly different copy. The three techniques agreed strongly for mortality and for the discharge models that react clearly to their inputs. The only weak case is the eICU discharge model, which is unusually “flat” (it reacts only weakly to any input), so the techniques agree on the main drivers (mean BP, comorbidities, age, and GCS) but disagree on the least important ones; for this model, the detailed ranking of the minor variables should be read with caution.

**Figure 7:**
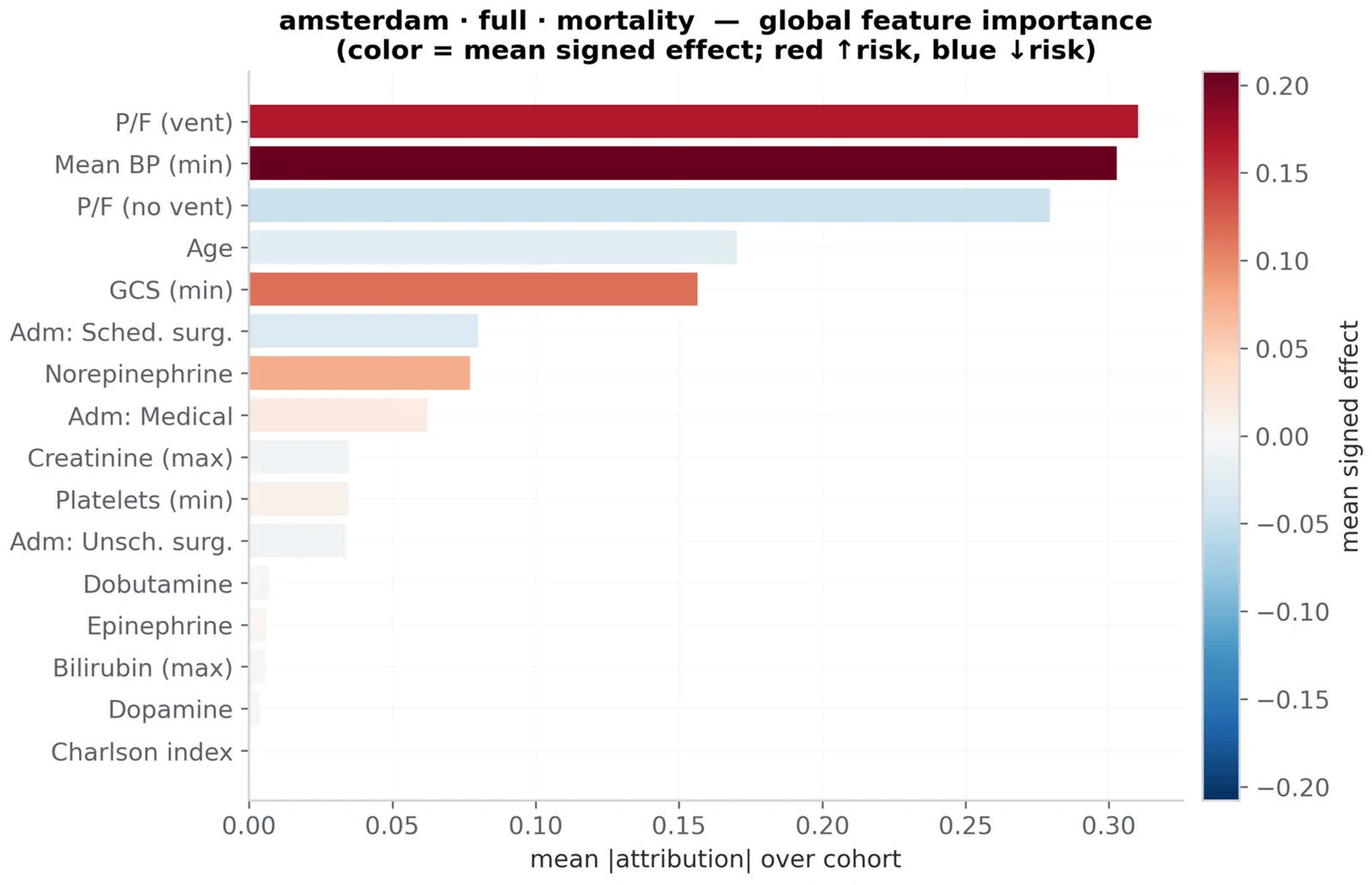
Cohort-level mortality driver importance for the Amsterdam full model (bar length is the mean attribution magnitude, and color is the mean direction, with red raising the risk). The equivalent figures for every database and task are provided in the project’s public repository (see the Data Availability Statement).

**Figure 8:**
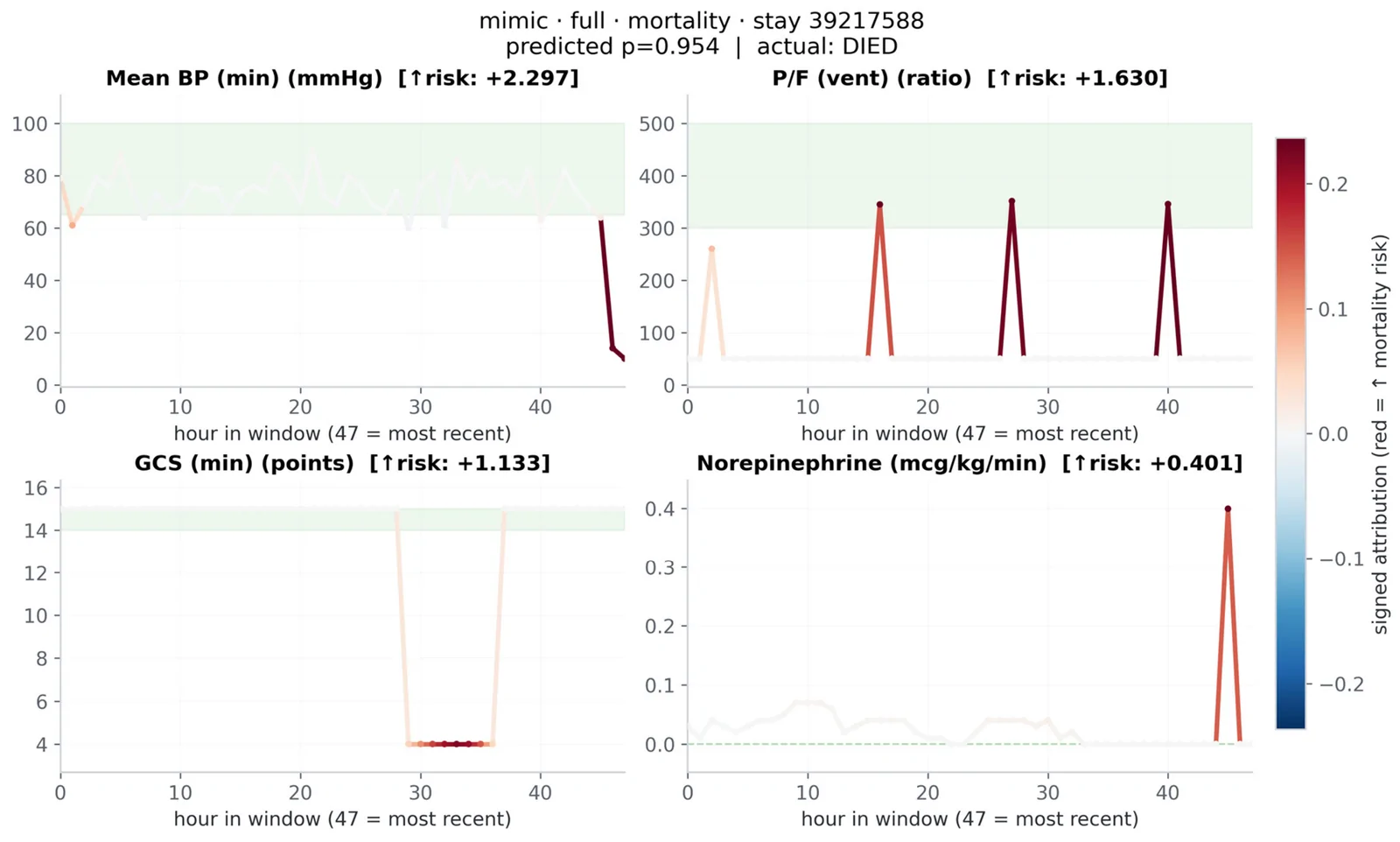
Per-patient drivers figure for a MIMIC patient who died (predicted risk 95%). Each panel shows a top driver’s actual measured values over time (line), colored red where that variable raised the risk and blue where it lowered it, with its normal range shaded in green. The late fall of mean BP below 65 mmHg drives the high-risk prediction.

**Figure 9:**
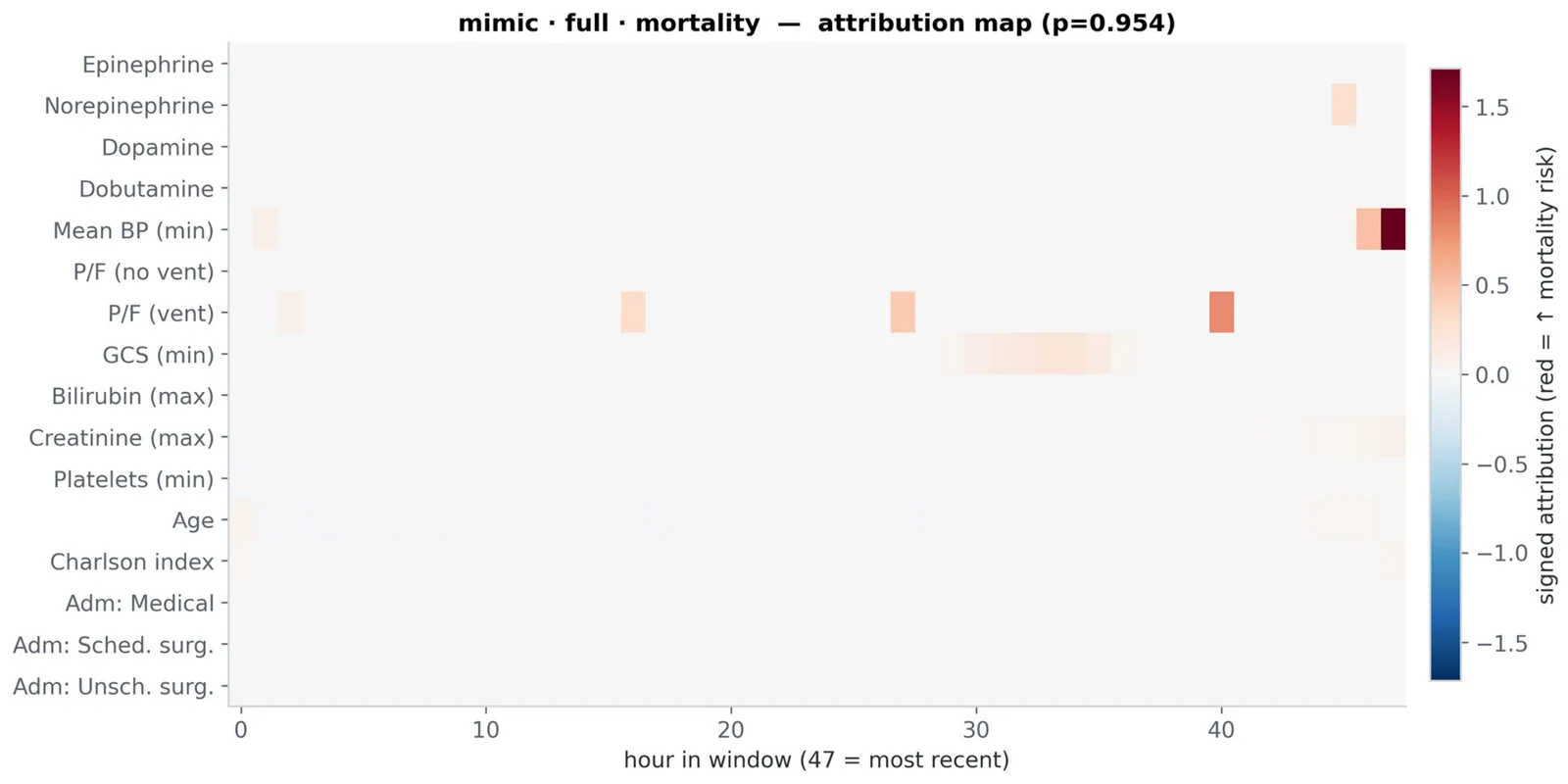
The full variable-by-hour map for the same patient (16 variables across the 48 h, with the most recent hour on the right). Red cells on the right indicate recent values that raised the predicted risk.

**Figure 10:**
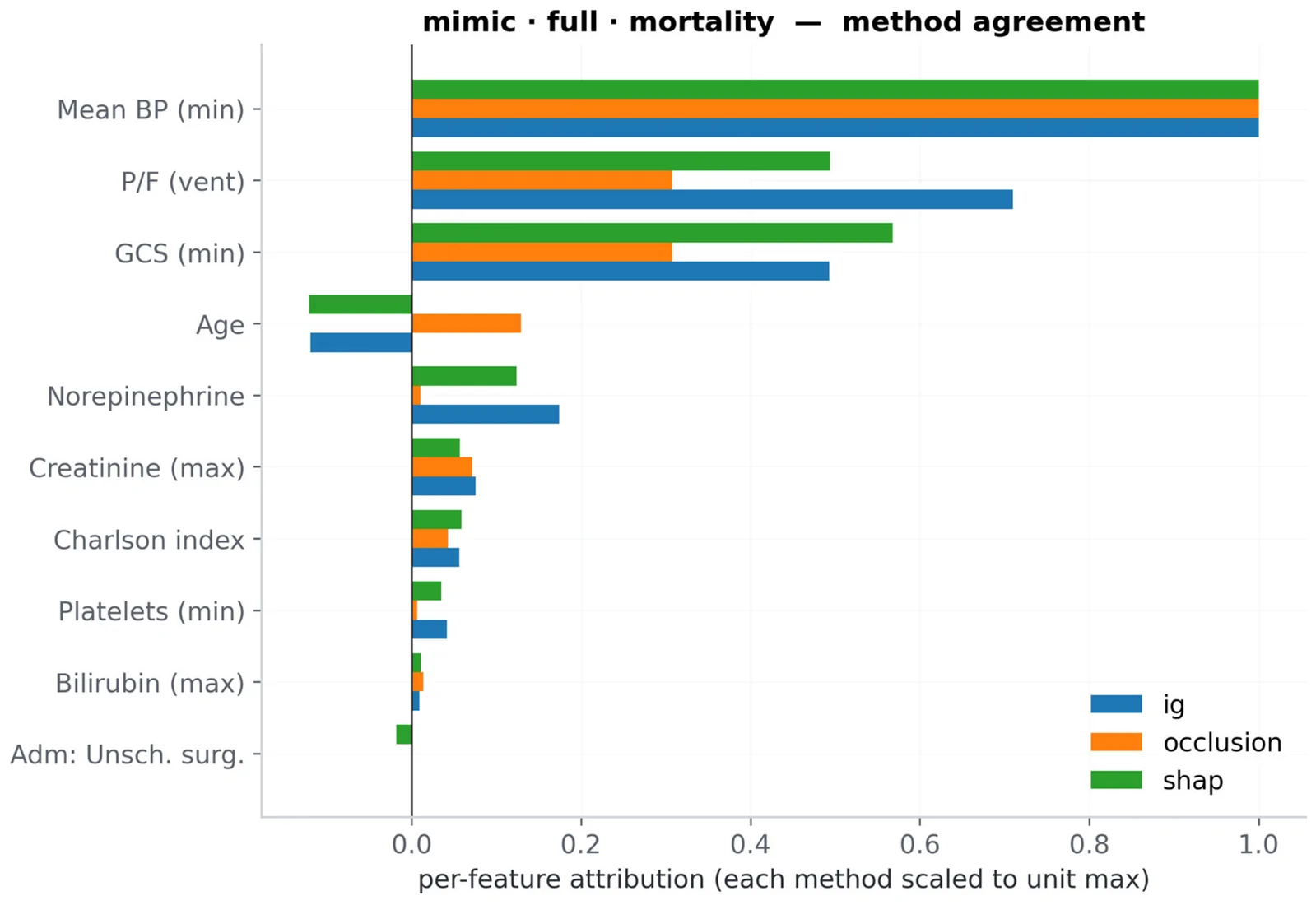
Agreement between the three explanation techniques (Integrated Gradients, Occlusion, and SHAP) for the same patient. When they point the same way for the most influential variables, this provides evidence of internal consistency of the explanation.

At the patient level, the layer produces the intended clinical narrative. For a representative MIMIC patient who died (predicted risk 95%), the main risk-raising drivers were a fall in mean arterial pressure from 75 to 52 mmHg, dropping below the 65 mmHg target, over the last hours, a falling ventilated P/F ratio, and norepinephrine. These appear in red, concentrated in the most recent hours, while the earlier in-range values are neutral. The same pattern appears in an eICU patient, with norepinephrine rising from 0.3 to 0.6 mcg/kg/min while the mean BP fell below 65 mmHg. For survivors, the same variables appear in blue, as in a MIMIC patient predicted at 1% risk, where an improving Glasgow Coma Score and a recovering mean BP lowered the risk. At the level of the whole unit, and as an exploratory observation not validated against a clinician reference standard, the most important variables make clinical sense and differ between hospitals: Amsterdam mortality relies most on mean BP, oxygenation (the ventilated P/F ratio), and the Glasgow coma score; eICU relies most on the type of admission and the Glasgow coma score; and HiRID relies most on the Glasgow coma score and mean BP. The models consistently pay most attention to the most recent hours. The cohort and per-patient outputs are shown in Figure 7, Figure 8, Figure 9 and Figure 10.

### 3.6. Calibration, Precision, and Operating-Point Performance

Discrimination (AUROC) does not by itself say whether the predicted probabilities can be trusted as numbers—which matters here, because the four-state visualization is produced by thresholding them—nor how the models behave at a usable decision threshold. For the locally adapted (full) models on each database we therefore also computed the area under the precision–recall curve (AUPRC), the Brier score, the calibration slope and intercept (a slope of 1 and an intercept of 0 indicate perfect calibration), and, at the threshold that maximizes sensitivity plus specificity, the sensitivity, specificity, and positive and negative predictive values (Table 3).

**Figure 11:**
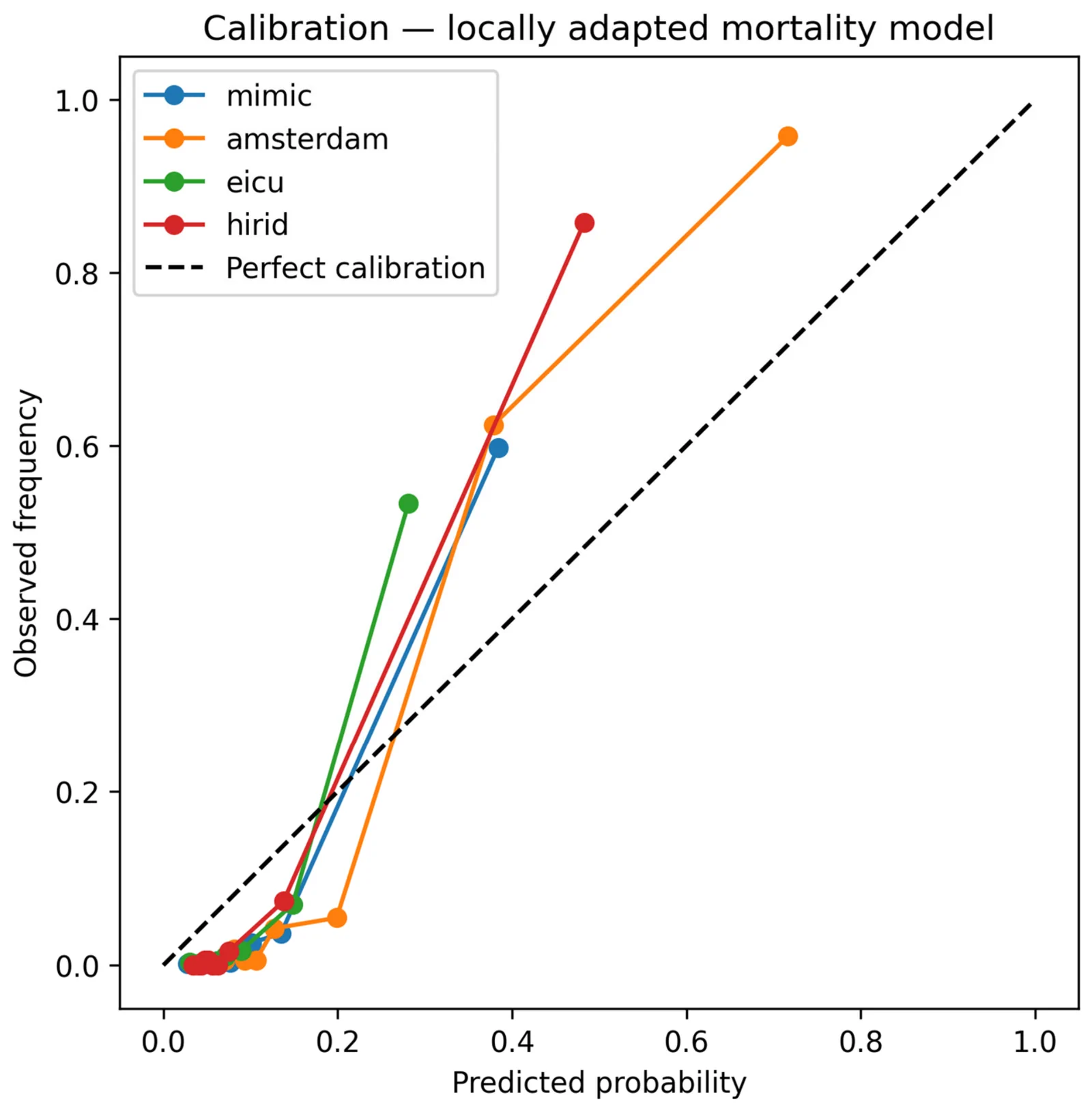
Calibration (reliability) curves for the locally adapted (full) mortality model on the four databases. A well-calibrated model lies on the diagonal; the curves show the compression and the systematic shift quantified in Table 3, which per-database recalibration corrects.

Two points stand out. First, the mortality models rank patients well—AUPRC 0.74–0.94, far above the 0.07–0.17 outcome prevalence, with negative predictive value at or above 0.99 on every database—but their probabilities are compressed and systematically shifted: the calibration slope is well above 1 (2.78–3.53) with a positive intercept everywhere, so the raw mortality probabilities are not calibrated out of the box and should be recalibrated per database (for example by Platt scaling) before they are mapped to the four-state display (Figure 11). Second, and by contrast, the discharge models are close to calibrated (slope near 1, intercept near 0) but discriminate less well (AUPRC about 0.75–0.79). In short, the discharge probabilities can be used much as they are, whereas the mortality probabilities need local recalibration; neither observation changes the discrimination reported in Table 2. The operating-point values use a threshold chosen on the test set and are therefore mildly optimistic; a fixed operating point should be set prospectively.

**Figure 12:**
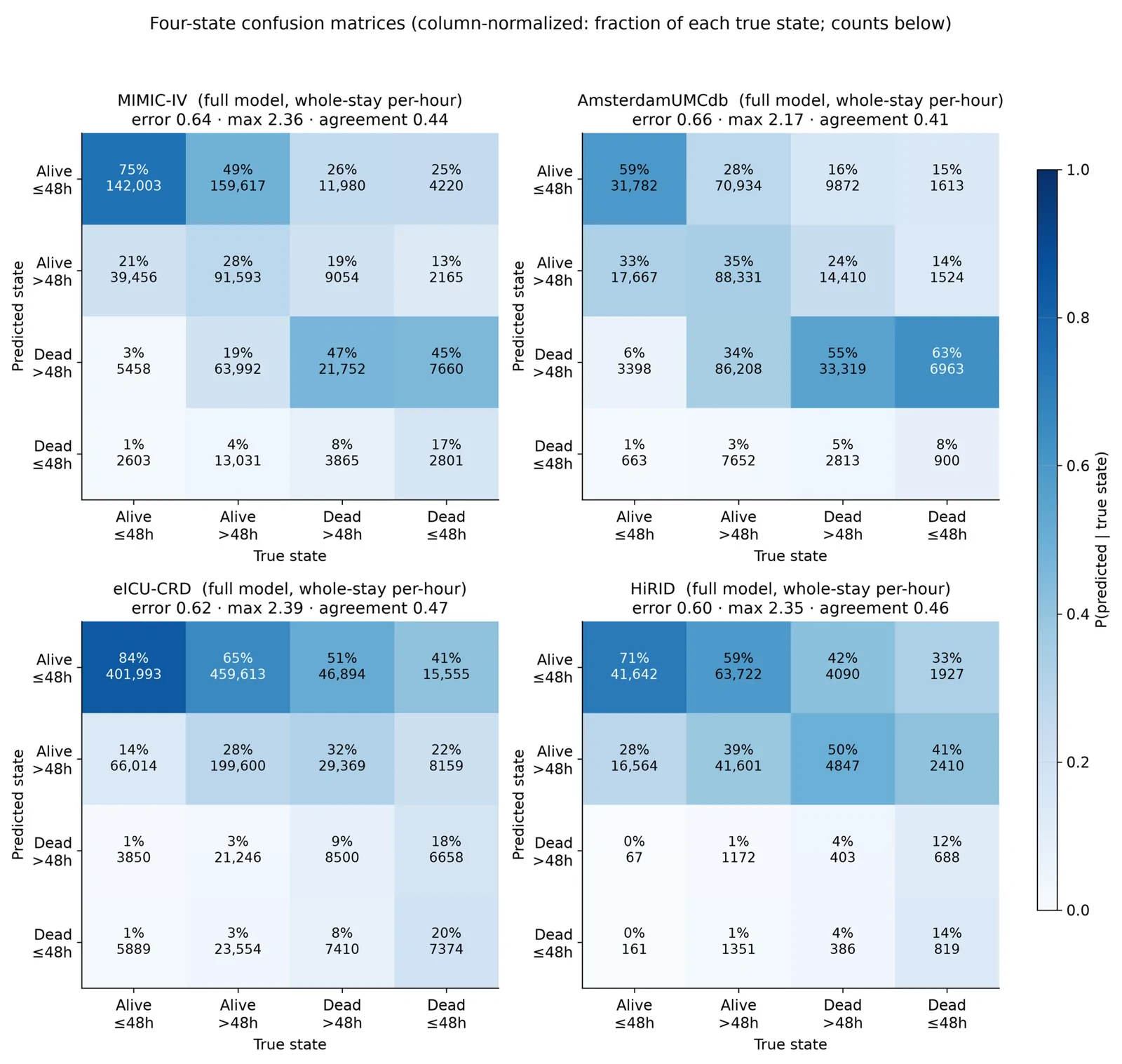
Four-state confusion matrices for the integrated PADS output on each database. Rows are the predicted state and columns the true state; the shading is the column-normalized share and the raw per-hour window counts are annotated. The four states combine the mortality and discharge predictions (alive and discharged within 48 h, alive and not discharged, dead within 48 h, and dead later). Most of the off-diagonal mass falls between the two survivor states, consistent with the weaker discharge model and the recalibration need noted above.

Although this study focuses on the two component models, we also checked the integrated four-state output that a clinician would see, using its custom error score [1] (0 is a perfect state assignment) computed directly from the inference outputs. The mean four-state error is similar across all four databases (0.64 on MIMIC, 0.66 on Amsterdam, 0.62 on eICU, and 0.60 on HiRID), against per-database maximum possible errors of 2.36, 2.17, 2.39, and 2.35, respectively; the first study reported a comparable error of 0.585 (maximum 2.387) on MIMIC. Exact agreement (the fraction of predictions whose four-state assignment exactly matches the true state) is 0.41–0.47 (against 0.25 expected by chance among four states). The full four-state confusion matrices (Figure 12), scored with the same severity weighting as the first study, show that most of the disagreement is between the two survivor states (alive and discharged within 48 h versus alive and not yet discharged), which is the discharge-timing split where the weaker discharge model and the harder early-stay mortality signal both blur; the death states are rarer and, on the uncalibrated and compressed mortality probabilities, tend to be over-assigned to the dead-later state. Because this uses the uncalibrated mortality probabilities, the recalibration above is expected to improve it, and we did not re-optimize the four-state thresholds here. This is a first external check of the combined product rather than only its two discriminators.

### 3.7. Mortality Discrimination as a Function of Lead Time Before the Outcome

**Figure 13:**
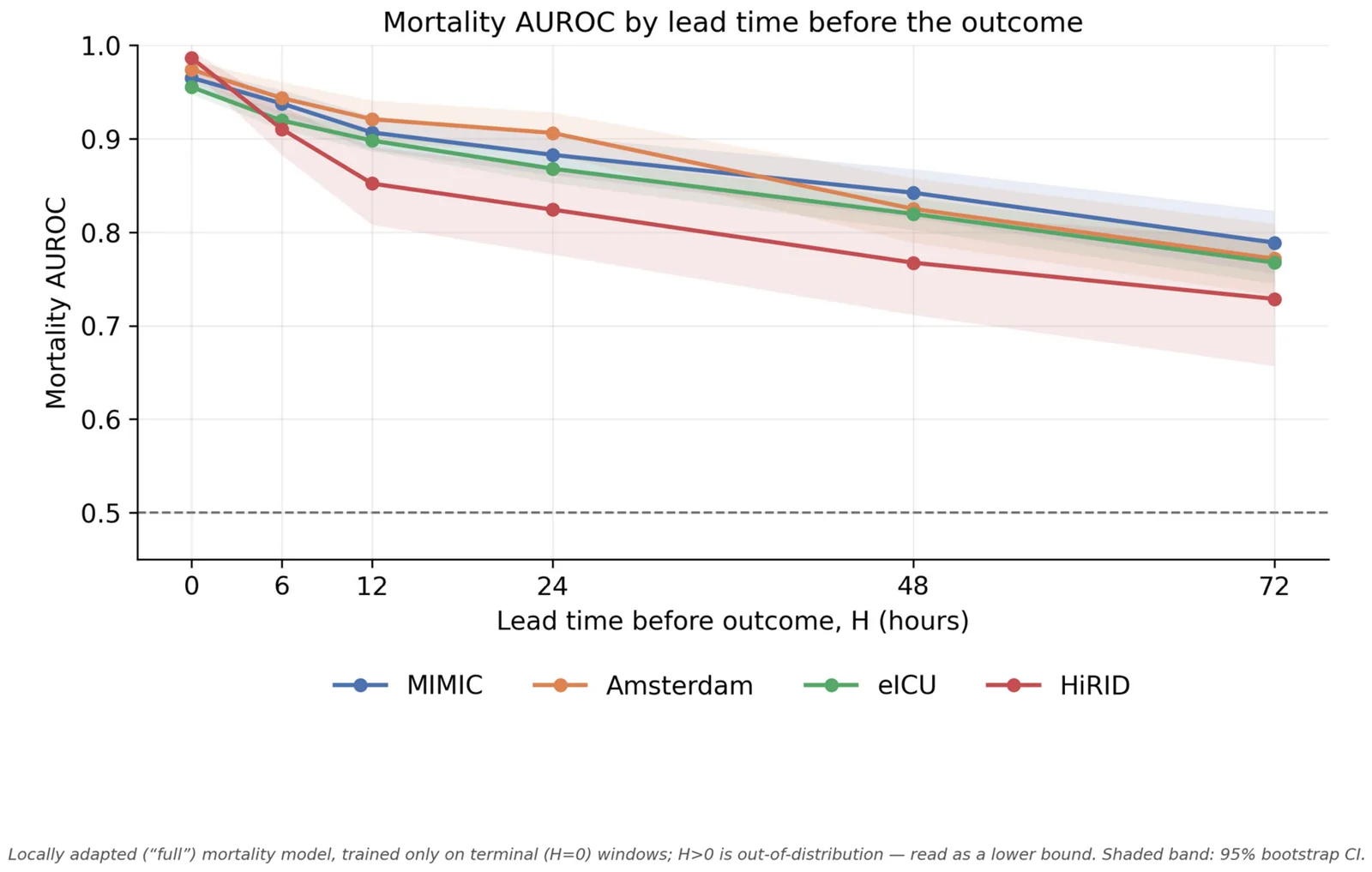
AUROC against the lead time before the outcome (H = 0, 6, 12, 24, 48, and 72 h) for the locally adapted mortality model, one line per database. Discrimination falls steadily with the lead time on the four databases; see Section 3.7 for the caveat on using the model outside the conditions it was trained for.

We re-evaluated the locally adapted (full) mortality models, without retraining them, at fixed lead times before the outcome: H = 0 (the terminal window already reported in Table 2) and 6, 12, 24, 48, and 72 h earlier. For the patients who died, the window is anchored on the time before death; for the survivors, it is anchored symmetrically on the time before their own ICU discharge, so both groups are measured relative to the end of their own stay. At H = 0, the analysis reproduces Table 2 exactly (6232 test stays on MIMIC, 1653 on Amsterdam, 15,567 on eICU, and 1830 on HiRID), which confirms that the two pipelines agree. Discrimination then falls steadily as the lead time grows, on every database (Table 4, Figure 13): MIMIC goes from 0.965 (H = 0) to 0.938 (H = 6) and 0.789 (H = 72), Amsterdam from 0.974 to 0.944 and 0.772, eICU from 0.955 to 0.920 and 0.768, and HiRID falls fastest and furthest, from 0.986 to 0.910 already at H = 6 and down to 0.729 at H = 72. Discrimination therefore remains substantial at clinically relevant horizons (AUROC 0.824 to 0.906 at 24 h and 0.767 to 0.842 at 48 h across the four databases), and it declines clearly only beyond that point. Because evaluating a longer lead time requires a longer stay, the eligible cohort shrinks as H grows (on MIMIC, for instance, 6232 stays at H = 0 against 2183 at H = 72; the counts for every database and horizon are given in Table 4), so the values at the largest H come from a subset of longer-staying patients and should be read with that in mind. Because this mortality model was trained only on terminal (H = 0) windows, applying it at H > 0 is an out-of-distribution test of a model never designed for early warning; the declining AUROC values at larger H should therefore be read as a lower bound on what a model explicitly trained for early prediction could achieve, not as evidence that the physiological signal predictive of death is absent earlier in the stay.

## 4. Discussion

This study addresses the three questions left open by the first PADS paper. The framework generalizes: across four ICU databases from different hospitals and countries, a small set of routinely collected variables supports strong mortality prediction (the highest values after local adaptation are between 0.955 and 0.986, terminal-window (near-outcome) discrimination) and useful discharge prediction (between 0.716 and 0.823). How well the model transfers, however, depends on the task, and this is the main contribution of the work. For mortality, the relationship between physiology and outcome is shared enough between hospitals that the MIMIC model is a good starting point: reusing it and retraining it locally gave the highest or near-highest discrimination on every database, and it removes the instability that affects training from scratch when deaths are rare, making the result reliable rather than dependent on a lucky run. For discharge, the MIMIC model transfers very little, and training a fresh model locally is as good as or better, because the timing of discharge depends on how each unit is run, not only on the patient’s physiology. These findings suggest the following task-specific deployment strategy: share and adapt the mortality model, and train the discharge model locally from scratch.

These findings are consistent with the federated approach that the first paper anticipated. Since reusing the mortality model both improves accuracy and makes training reliable, our results point towards a federated arrangement in which a network of ICUs would share a common mortality model and adapt it locally, without ever sharing patient records, so that each hospital could contribute improvements while its data stay in-house. We did not test a full federated setup here; what our experiments establish is the building block such an arrangement relies on, namely that the mortality model parameters learned in one setting can provide a useful initialization for adaptation to another setting. This is the situation where reusing a shared model, rather than each hospital training on its own, is an advantage. The fact that discharge is better trained locally is not a weakness of this scheme but a clarification of it, since only the physiologically driven outcome (mortality) needs to be shared. A genuine federated model, trained across several ICUs without pooling their data, is a natural next step (Section 5).

The explainability layer removes a barrier that the first study had stated explicitly. By producing a reliable, time-based explanation, and by relying on three techniques that are cross-checked rather than on a single one, it provides a clinically interpretable representation of the factors contributing to each prediction (for example, “the risk rose because the mean BP fell below ~65 mmHg in the last 12 h while the patient was on norepinephrine”) and lets clinicians see, and question, what each model relies on, both for an individual patient and across the whole unit. The three techniques are computationally light and can be run at any point in the stay; here we generated explanations at the most decision-relevant moment, but the same output could equally support a real-time bedside display or a post-discharge debrief. It is important to take into account that the tool reports what the model does. Any anomalous explanation is a finding about the model, not necessarily a clinical truth, and the checks conducted only confirm that the explanation faithfully reflects the model. This distinction is important for a safe interpretation, and a disclaimer to this effect should be included when the tool is put into production.

### 4.1. Comparison with Prior External Validation

The distinction between the two tasks also refines the closest prior work. Lim et al. [5] externally validated a frozen short-term-mortality model and reported AUROCs of about 0.89 on MIMIC, 0.89 on eICU, and 0.87 on AmsterdamUMCdb, without local adaptation. Our frozen (direct-transfer) mortality model behaves comparably on the databases closest to its development cohort (eICU 0.834, HiRID 0.868) but collapses on the most divergent one (Amsterdam 0.489); after local adaptation, it reaches 0.955–0.986. Read together, this suggests that a single frozen model can travel reasonably on average, but that local adaptation is what makes mortality prediction reliable on a genuinely different unit. Unlike that work, we also release reproducible code, an explicit transfer comparison, and a bedside explanation layer. The added value of the temporal model over simpler alternatives was already established in the first study, where PADS mortality prediction outperformed the standard ICU severity scores (APACHE III, OASIS, and SAPS II) across length-of-stay bins [1]; we therefore did not repeat that baseline comparison here.

### 4.2. What the Mortality Score Means Before the Terminal Window

The analysis by lead time in Section 3.7 shows that mortality discrimination is high only near the end of the stay (Table 4), which raises the question of what the mortality score represents away from that terminal window. It could be a literal, time-varying probability of dying soon, or it could work more like a physiological severity score, that is, a measure of how closely the current physiology of a patient resembles that of previous non-survivors, regardless of how soon death is expected. Two supplementary analyses, run on every 48-h window of the whole stay for the four databases and separately from the main evaluation pipeline reported above, support the second reading. First, the mean mortality score of the patients who eventually die is already high in the earliest windows available and stays roughly flat over the following one to two weeks, instead of climbing as death approaches. Second, even in early windows (48–72 h after admission), the score correlates with the concurrent organ-dysfunction burden (a SOFA proxy computed from the same clinical variables), with r between 0.29 and 0.72 across the four databases (highest on eICU and lowest on Amsterdam). Both findings fit a severity score better than a literal short-term probability, which would be expected to rise towards the end of the stay. They also fit the compressed-probability calibration pattern reported in Section 3.6: a network trained mainly to separate severe patients from milder ones, rather than to follow the timing of the outcome, would be expected to discriminate well while its raw output drifts away from a calibrated probability scale. This also explains the shape of Table 4. If the score of the patients who die is already high early and does not climb, the rise in discrimination towards H = 0 comes largely from the survivors, whose scores fall as they recover and approach their own discharge. Terminal-window discrimination is therefore high because it contrasts a deteriorating patient with one who is close to leaving the unit, which is the most favorable comparison available in the stay.

This reading describes better what the mortality network encodes, but it does not recover the ability of the panel to anticipate death with a useful lead time, and it should not be presented as if it did. It could be expected that the timing dimension of the discharge network compensates for this, so that the combined four-state panel still moves to the dead-within-48-h state some time before death even when the mortality score is not rising. Under the thresholds used here, for the most part it does not. For each patient who died, we looked for the last point at which the predicted state switched to dead within 48 h and stayed there for the rest of the stay. In 54–85% of these patients, depending on the database, that switch never happens: the predicted state remains dead later for the whole stay, including the final 48 h. In the minority of patients in whom it does happen, the median lead time is only 1–4 h. This is the pattern already visible in Figure 12, where the dead-later state absorbs most of the true dead-within-48-h windows (exact agreement 0.41–0.47); the individual patient trajectories show that this over-assignment is concentrated in a large subset of the patients who die, whose predicted state never changes at all, rather than being spread evenly across them.

Two considerations bound how this should be read. First, it is a property of the state mapping rather than of the mortality model itself: at the operating point reported in Table 3, which is chosen on the test set and is therefore mildly optimistic, the same model identifies 89–95% of the deaths in the terminal window, so what fails here is the translation of a probability into a discrete state, not the ranking of the patients. Second, and for the same reason, the four-state assignment is produced by fixed thresholds applied to the uncalibrated, compressed probabilities described in Section 3.6, and we did not recalibrate them or re-optimize the thresholds for this analysis. Whether the per-database Platt recalibration set out in Section 4.4, together with thresholds re-fitted on the recalibrated scale, would recover a useful lead time is an open question that these results do not answer, and it is the most direct next step for the integrated panel.

One factor partly compensates for the missing lead time. Unlike the mortality network, the discharge network is validated across the whole stay (roughly 90–229 windows per stay), so the discharge-timing half of the panel does not carry the terminal-window caveat. This narrows the gap, but it does not close it. Until the mortality half has comparable support, we recommend presenting the mortality dimension to clinicians as a running severity indicator, useful for situational awareness at any point in the stay, rather than as a signal that death is imminent.

### 4.3. Intended Use at the Bedside

PADS is designed to support passive risk stratification rather than active decision support. The framework does not generate clinical alerts, and it does not trigger any automated action, escalation, or de-escalation of care. Instead, the four-state panel (alive and discharged within 48 h, alive and not discharged, dead within 48 h, dead later) is intended to be visible to the clinical team during rounds, alongside the standard chart, as one additional piece of situational information rather than as a directive. Predictions are updated every hour as new measurements arrive, which is the frequency at which the underlying variables are themselves recorded; this keeps the panel up to date without implying a finer warning capability than the results of Section 3.7 support. Within this passive design, we see three concrete uses: supporting discharge and step-down planning by flagging patients whose predicted probability of discharge within 48 h is rising; supporting unit-level bed and staffing planning by providing a real-time summary of the unit’s four-state distribution; and helping the clinical team decide which patients to review first during rounds, without substituting for that review. Any use of the PADS output to trigger or directly inform escalation or de-escalation decisions, as opposed to this passive, informational role, would require prospective validation of clinical impact before it could be recommended, for the reasons discussed in Section 4.5.

### 4.4. Recalibration in Practice

Because the locally adapted mortality models discriminate well but produce compressed and systematically shifted probabilities (calibration slope between 2.78 and 3.53, with a positive intercept on all four databases; Section 3.6), recalibration would be required before the four-state panel could be considered ready for clinical use, and it is worth saying what that would involve in practice. The most practical approach for routine deployment is Platt scaling (logistic recalibration), that is, a single one-dimensional logistic regression fitted on top of the output score of the frozen model, estimated on a retrospective local cohort covering some 6 to 12 months of the admissions of the centre itself. This step would be done by the data or informatics team of the centre itself as part of the deployment, and not by the developers of the model, since it only requires predictions and outcomes that are already held locally, and no access to the underlying network, its weights, or its training data. Calibration should not be assumed to remain stable once deployed: case mix, treatment practice, and coding conventions can drift over time, so we recommend quarterly monitoring of the calibration slope, intercept, and observed-to-expected (O/E) mortality ratio on a rolling recent cohort, with re-fitting of the Platt-scaling parameters triggered whenever any of these drift outside a predefined tolerance (for example, a slope departing from 1 by more than a fixed margin, or an O/E ratio outside a 0.8–1.25 range). It is important to take into account what recalibration does and does not change: it maps the output probabilities of the model onto the observed frequencies of the outcome through a monotonic transformation, and leaves the weights of the network, and therefore the discrimination and the ranking of the patients, untouched. This is what makes recalibration cheap to implement and easy to audit (a two-parameter fit and a handful of summary statistics, which the local staff can review without access to the model itself), in contrast to retraining the network, which would only be justified if what degraded were the discrimination rather than the calibration.

### 4.5. Performance, Calibration, Interpretability, and Utility Are Distinct

The results reported above do not establish that PADS is ready to guide decisions at the bedside, and it is worth separating the different kinds of evidence that a clinical prediction tool needs before such a claim can be made. Discrimination (Section 3.1, Section 3.2, Section 3.3 and Section 3.4), calibration (Section 3.6), interpretability (Section 3.5), and clinical utility are four different properties, and good evidence for one of them does not carry over to the others.

The agreement between Integrated Gradients, Occlusion, and SHAP (Section 2.6 and Section 3.5) shows that three techniques that estimate the contribution of the variables in different ways arrive at a similar account of what the model is doing; together with the automatic checks described in Section 2.6, this is evidence of internal consistency and of fidelity to the model. It is not evidence that the variables identified are clinically causal, nor that the mechanism the model has learned corresponds to the underlying physiology of the patient. As stated above, the tool reports what the model does; a consistent explanation describes the reasoning of the model faithfully, but it does not by itself describe a fact about the patient.

The same separation applies to calibration and clinical utility. A model can discriminate well, be correctly calibrated after the procedure described above, and be faithfully explained by the three techniques, and none of that establishes that acting on its output improves outcomes. A well-calibrated and faithfully explained probability is informative; whether using it at the bedside to escalate, de-escalate, or reallocate care actually changes what happens to the patients is a separate question, and one that a retrospective evaluation based only on databases, such as this one, cannot answer. Answering it would require a prospective study of clinical impact, ideally comparing care with and without the PADS panel available to the clinical team against predefined endpoints, before the framework could be considered ready for routine use at the bedside.

### 4.6. Limitations

Several limitations should be considered when interpreting these findings. First, mortality discrimination is measured on the final 48-h window of the stay: this is terminal-window (near-outcome) discrimination, an easier and less anticipatory task than early warning, so the high mortality AUROC values should be read in that light; a whole-stay comparison is given in Appendix C (Table A2), and it confirms that whole-stay mortality discrimination is substantially lower than at the terminal window. A more detailed analysis by lead time is given in Section 3.7 (Table 4, Figure 13); in that analysis the survivors are anchored on their own discharge time rather than on a death time, which keeps the two groups symmetric, each anchored at the end of its own stay, but since discharge timing is not itself random, some residual selection bias in the survivor group may remain. Second, the discharge confidence intervals are too narrow because each patient contributes several correlated time windows; the discharge point values are reliable, but the intervals are only indicative, and we therefore compare discharge training strategies with a stay-clustered test (Section 3.4). Third, the locally adapted mortality models discriminate well but are not calibrated out of the box, since their probabilities are compressed and systematically shifted (Section 3.6); because the four-state visualization is produced by thresholding probabilities, the mortality probabilities must be recalibrated per database before that mapping.

Fourth, the train/test splits are by stay rather than grouped by hospital: for the multicenter eICU database this means the retrained result reflects within-hospital deployment (a unit retraining on its own patients and applying the model to its own future patients) rather than generalization to entirely new hospitals, which would require a leave-hospital-out split, whereas the direct-transfer eICU result (0.834) uses no eICU training data and is unaffected; relatedly, because some patients have more than one stay, the by-stay split can place different stays of the same patient in train and test, a potential patient-level information leakage we did not control for and which affects all retrained conditions.

Whether this affected the results was checked on the two databases where a patient identifier could be recovered, MIMIC and Amsterdam, using the models already trained and without retraining anything. On MIMIC, 31.50% of the test stays shared a patient with the training set; removing them (leaving 4269 of the 6232 test stays) left the results essentially unchanged for both tasks, with mortality going from 0.9652 to 0.9675 and discharge from 0.7420 to 0.7533. In Amsterdam, 18.15% of the test stays overlapped by patient; removing them (leaving 1353 of the 1653 test stays) again left the results essentially unchanged, with mortality going from 0.9741 to 0.9766 and discharge from 0.8148 to 0.8245. In both databases, the values moved slightly upwards rather than downwards once the overlapping stays were removed, and every difference falls well inside the 95% bootstrap interval of the corresponding unfiltered value. This argues against same-patient overlap inflating the reported performance, since if leakage were driving the results, the values should have dropped rather than held or risen; we do not read the small rise as an improvement produced by removing the leakage, because it is more plausibly explained by the smaller, and therefore noisier, filtered test set. The check could not be extended to the other two databases. In the case of eICU, the field that links several stays to the same patient (patienthealthsystemstayid) was queried during the original extraction but was not kept in the final processed dataset, and re-extracting it was not possible here, so this is a limitation of the check itself rather than evidence that eICU is free of this form of leakage. In the case of HiRID, no patient identifier distinct from the per-admission stay identifier exists in the available data, which is a feature of the public release rather than a gap in our extraction, so the check could not be done at all, and patient-level leakage there remains an unquantified caveat.

Fifth, on MIMIC, the comparison between the shipped model used directly and the retrained model is within-sample, since the shipped model had already seen the current MIMIC test sets; any such overlap can only inflate the lower (direct-transfer) value and so cannot account for the retraining gain, and it does not affect the external databases. Sixth, HiRID was missing 5 of the 16 variables, which were set to constants; this only affects the direct-transfer condition, so the HiRID value of 0.868 there should be read with that caveat. Seventh, the conditions that start from the MIMIC model were each run once, after we confirmed on one of them that the result barely changes with the random starting point; only the unstable from-scratch condition was repeated across several seeds. Eighth, the explanations were produced for the well-performing locally adapted models only; their cohort-level patterns are exploratory rather than validated against a clinician reference standard, agreement among the three techniques is an internal-consistency check rather than a proof of faithfulness, and for the eICU discharge model the detailed ranking of the minor variables is less reliable. Ninth, discharge is intrinsically harder to predict than mortality in every database, reflecting the non-physiological factors that drive discharge timing, and the discharge results (around 0.7 to 0.82) leave room for improvement; adding variables that describe how the unit is run would likely help. Tenth, as in the first study, this is a retrospective evaluation based only on databases, and a prospective validation in a real unit, with clinicians, is still future work. Finally, we did not rely on the performance values reported automatically during training, because in some settings these can look good even for a model that does not actually discriminate well; instead, we recomputed every value with an independent calculation, and we recommend that similar studies do the same.

## 5. Conclusions

We tested the PADS mortality and discharge framework on four different ICU databases and characterized how well it transfers between them. Transportability proved to be task-dependent: what the mortality model had learned about physiology transferred between hospitals, whereas what the discharge model had learned about the timing of discharge did not. The mortality model benefits from being trained elsewhere first and then adapted locally, which provided the highest discrimination in this study and, by stabilizing training when deaths are rare, a more robust initialization than training from scratch (see Section 3.3 for limitations on this conclusion: we did not test whether alternative initializations or resampling would remove the instability equally well). The mortality values behind this conclusion are terminal-window discrimination, measured on the final 48 h of the stay; as Section 3.7 shows, discrimination falls steadily as the lead time before the outcome grows, so the framework should not be read as an early-warning model. The discharge model, which is also driven by how each unit is organized, is better trained locally from scratch. This task-dependent recommendation, together with the stabilizing effect of reusing a shared model, supports a federated approach in which the mortality model is shared and adapted across hospitals while each hospital trains its own discharge model. Finally, a clear explainability layer with internal consistency checks provides an interpretable representation of model predictions that may facilitate bedside assessment and lets clinicians audit what each model relies on, addressing the main barrier to adoption that was identified in the first study. Future work will focus on prospective validation in a real unit, federated training across collaborating ICUs, better confidence intervals for discharge together with variables describing how the unit is run, and recovering the variables that were missing in HiRID.

## Figures and Tables

**Table 1:** External-validation cohorts. Mortality is evaluated once per stay (the final 48 h window), while discharge is evaluated over several 48 h anchor windows per stay, so the number of discharge evaluation units is larger. Mortality prevalence is the proportion of test stays that died in the ICU.

Database	Country	Total Stays	Train Stays	Test Stays	Discharge Windows (Test)	Mortality Prevalence (Test)
MIMIC-IV (source) ^1^	USA (Boston)	31,160	24,928	6232	22,160	~7%
AmsterdamUMCdb	Netherlands	8262	6609	1653	6760	~17%
eICU-CRD	USA (multicenter)	77,832	62,265	15,567	54,958	~6.5%
HiRID	Switzerland	9150	7320	1830	6795	~9.3%

^1^ The MIMIC-IV stay count differs from the first PADS paper (32,875 stays) because the re-implemented pipeline used here excludes 1715 stays whose ICU mortality outcome was missing (32,875 − 1715 = 31,160), whereas the first paper kept them. The two cohorts are otherwise identical: they start from the same data extract and apply the same minimum length-of-stay of 48 h.

**Table 2:** External-validation AUROC by database and condition. For scratch, mortality is reported as median [min, max] over the seeds (4–5 per database) because it is bimodal—a minority of random initializations collapse to the base rate—whereas the seed-stable scratch discharge is the mean ± SD; all other cells are single-seed point estimates with 95% bootstrap intervals. The discharge intervals are row-level and therefore too tight (Section 2.5). HiRID direct transfer uses 5 constant-imputed inputs (Section 2.3). Bold marks the best mortality condition per database; discharge conditions are not bolded because the differences between training strategies are within noise once the within-stay correlation is accounted for (Section 3.4).

Database	Condition	Mortality AUROC	Discharge AUROC
MIMIC (same database)	Direct transfer	0.942 [0.928, 0.956]	0.716 [0.709, 0.723]
	Scratch	0.933 [0.610, 0.939]	0.727 ± 0.008
	Full	**0.965 [0.954, 0.975]**	0.742 [0.735, 0.749]
	Dense	0.953 [0.940, 0.965]	0.730 [0.724, 0.737]
	LSTM	**0.965 [0.954, 0.976]**	0.742 [0.735, 0.749]
Amsterdam	Direct transfer	0.489 [0.454, 0.527]	0.651 [0.638, 0.664]
	Scratch	0.963 [0.953, 0.967]	0.823 ± 0.025
	Full	**0.974 [0.963, 0.984]**	0.815 [0.805, 0.825]
	Dense	0.926 [0.905, 0.947]	0.807 [0.796, 0.817]
	LSTM	0.973 [0.960, 0.984]	0.804 [0.793, 0.814]
eICU	Direct transfer	0.834 [0.819, 0.849]	0.552 [0.547, 0.557]
	Scratch	0.950 [0.773, 0.951]	0.716 ± 0.002
	Full	**0.955 [0.947, 0.963]**	0.698 [0.693, 0.702]
	Dense	0.931 [0.921, 0.940]	0.676 [0.671, 0.680]
	LSTM	**0.957 [0.950, 0.964]**	0.687 [0.682, 0.692]
HiRID	Direct transfer	0.868 [0.829, 0.902]	0.570 [0.556, 0.584]
	Scratch	0.971 [0.714, 0.978]	0.759 ± 0.007
	Full	**0.986 [0.975, 0.995]**	0.734 [0.722, 0.746]
	Dense	0.977 [0.965, 0.987]	0.703 [0.692, 0.716]
	LSTM	0.975 [0.957, 0.990]	0.732 [0.719, 0.744]

**Table 3:** Calibration, precision–recall, and operating-point performance of the locally adapted (full) models on each database’s held-out test set. Operating-point metrics (Sens, Spec, PPV, NPV) are at the sensitivity-plus-specificity-maximizing threshold. Perfect calibration is slope 1, intercept 0.

Database	Task	AUROC	AUPRC	Brier	Cal. Slope	Cal. Int.	Sens	Spec	PPV	NPV
MIMIC	Mortality	0.965	0.862	0.034	3.53	3.52	0.90	0.94	0.51	0.99
Amsterdam	Mortality	0.974	0.914	0.056	2.78	1.74	0.93	0.94	0.77	0.99
eICU	Mortality	0.955	0.740	0.042	3.21	3.24	0.89	0.92	0.43	0.99
HiRID	Mortality	0.986	0.940	0.034	3.28	3.18	0.95	0.96	0.72	0.99
MIMIC	Discharge	0.742	0.767	0.198	0.99	0.37	0.83	0.56	0.73	0.69
Amsterdam	Discharge	0.815	0.734	0.171	0.99	−0.23	0.82	0.72	0.69	0.84
eICU	Discharge	0.698	0.748	0.217	1.01	0.42	0.74	0.57	0.73	0.59
HiRID	Discharge	0.734	0.760	0.209	0.81	0.26	0.73	0.63	0.71	0.65

**Table 4:** Mortality AUROC of the locally adapted (full) model as a function of the lead time (H, in hours) before the outcome, which is death for the patients who died and their own ICU discharge for the survivors, for each database, with 95% bootstrap intervals and the eligible cohort size (*n*, and *n* deaths) at each H. H = 0 reproduces Table 2.

Database	H (Hours)	AUROC	95% CI	*n*	*n* Deaths
MIMIC	0	0.965	[0.954, 0.975]	6232	430
MIMIC	6	0.938	[0.923, 0.952]	5511	407
MIMIC	12	0.907	[0.888, 0.924]	5085	387
MIMIC	24	0.883	[0.861, 0.903]	4129	351
MIMIC	48	0.842	[0.815, 0.868]	2872	276
MIMIC	72	0.789	[0.756, 0.823]	2183	225
Amsterdam	0	0.974	[0.962, 0.984]	1653	284
Amsterdam	6	0.944	[0.926, 0.961]	1546	266
Amsterdam	12	0.921	[0.898, 0.941]	1481	243
Amsterdam	24	0.906	[0.883, 0.928]	1308	227
Amsterdam	48	0.825	[0.789, 0.858]	1097	189
Amsterdam	72	0.772	[0.733, 0.809]	956	168
eICU	0	0.955	[0.948, 0.963]	15,567	1012
eICU	6	0.920	[0.910, 0.929]	13,879	945
eICU	12	0.898	[0.887, 0.909]	12,724	875
eICU	24	0.868	[0.853, 0.881]	10,196	768
eICU	48	0.820	[0.802, 0.836]	7073	599
eICU	72	0.768	[0.745, 0.789]	5144	489
HiRID	0	0.986	[0.974, 0.995]	1830	170
HiRID	6	0.910	[0.883, 0.935]	1650	153
HiRID	12	0.852	[0.808, 0.891]	1560	139
HiRID	24	0.824	[0.776, 0.868]	1291	119
HiRID	48	0.767	[0.712, 0.820]	951	91
HiRID	72	0.729	[0.657, 0.793]	721	70

## Data Availability

All four databases are publicly available to credentialed researchers: MIMIC-IV and eICU-CRD through PhysioNet, AmsterdamUMCdb through the Amsterdam Medical Data Science initiative, and HiRID through PhysioNet. The transfer-learning sweep, the multi-seed stability study, the bootstrap analysis, the figures, and the explainability package, together with the ETL adapters, are released in the project’s public repository to allow reproduction and external validation. Access to the underlying databases must be requested from the respective providers.

## Appendix A. Per-Variable Harmonization Across Databases

Each of the 16 model inputs was mapped to a source field and unit in every database, reproducing the MIMIC-IV reference schema. Cells give the source (and, where converted, the target unit). The HiRID source fields for the data-derived variables are carried from a preprocessed extract that maps them to the upstream HiRID variables (raw pharmaid, variableid, and observation identifiers); the HiRID constant-imputed cells are also shown.

**Feature (Target Unit)**	**MIMIC-IV**	**AmsterdamUMCdb**	**eICU-CRD**	**HiRID**
rate_epinephrine (mcg/kg/min)	mimic_derived.sofa	drugitems 6818, gamma → mcg/kg/min	infusiondrug (epinephrine) → mcg/kg/min	Givendose (pharmaid 71, 1000649, 1000650, 1000655, 1000750) → mcg/kg/min
rate_norepinephrine (mcg/kg/min)	mimic_derived.sofa	drugitems 7229 → mcg/kg/min	infusiondrug (norepinephrine) → mcg/kg/min	Givendose (pharmaid 1000462, 1000656, 1000657, 1000658) → mcg/kg/min
rate_dopamine (mcg/kg/min)	mimic_derived.sofa	drugitems 7179 → mcg/kg/min	infusiondrug (dopamine) → mcg/kg/min	constant 0.0 (absent)
rate_dobutamine (mcg/kg/min)	mimic_derived.sofa	drugitems 7178 → mcg/kg/min	infusiondrug (dobutamine) → mcg/kg/min	Givendose (pharmaid 426) → mcg/kg/min
meanbp_min (mmHg)	mimic_derived.sofa	numericitems 6642/6679/8843, hourly MAX of MAP	invasive + non-invasive MAP, hourly MIN	Observation variableid 110, 610 hourly MIN
pao2fio2ratio_novent (mmHg)	mimic_derived.sofa	PaO_2_/FiO_2_ when not ventilated	resp. P/F when mechvent = 0	Average PaO_2_ × 100/average FiO_2_ when is_ventilated = 0
pao2fio2ratio_vent (mmHg)	mimic_derived.sofa	PaO_2_/FiO_2_ when ventilated	resp. P/F when mechvent = 1	Average PaO_2_ × 100/average FiO_2_ when is_ventilated = 1
gcs_min (3–15)	mimic_derived.sofa	reconstructed from GCS components, hourly MIN	physicalexam + nursecharting GCS, MIN	Verbal GCS, Motor GCS and Eye GCS. MIN
bilirubin_max (mg/dL)	mimic_derived.sofa	numericitems 6813/9945, µmol/L ÷ 17.1	lab total bilirubin, MAX	Bilirubin MAX, µmol/L ÷ 17.1
creatinine_max (mg/dL)	mimic_derived.sofa	numericitems 6836/9941/14,216, µmol/L ÷ 88.4	lab creatinine, MAX	Creatinine MAX, µmol/L ÷ 88.4
platelet_min (×10^9^/L)	mimic_derived.sofa	numericitems 9964/6797/10,409/14,252, MIN	lab platelets, MIN	Platelets MIN
admission_age (years)	icustay_detail.admission_age	agegroup band midpoint	patient.age (‘>89’ → 89)	General table, Age
charlson_comorbidity_index	mimic_derived.charlson	not available (left missing/uninformative)	derived from pasthistory (missing → 0)	constant 5.78 (MIMIC mean)
admission_type_Medical	rule on admission type + surgical	specialty + urgency rule	default when not surgical	constant 1.0
admission_type_ScheduledSurgical	elective & surgical	surgical & urgency = 0	operative & elective	constant 0.0
admission_type_UnscheduledSurgical	non-elective & surgical	surgical & urgency = 1	operative & emergent	constant 0.0
Discharge outcome (disch_48 h ground truth)				
Discharge field (end of ICU unit stay)	icustay_detail.outtime	admissions.dischargedat	patient.unitdischargeoffset	dischargetime (via window_id)
ICU death flag (icu_expire_flag) source	Derived: dod (date-only) same day as/before outtime (vi)	Native: destination = ‘Overleden’	Native: unitdischargestatus = ‘Expired’	Native: discharge_status == ‘dead’ (Exitus) (viii)
Hospital-level mortality flag (hospital_expire_flag)	Native, independent field	Copied from icu_expire_flag (vii)	Native, independent field	Not present in schema
Death excluded from discharge/disch_48 h timeline?	No	No	No	No
Ward/step-down transfer distinguished from final discharge?	No, any destination ends the stay record identically	No, folded into “discharge alive”	No, same as MIMIC	Not fully determinable from available code
Cross-stay readmission stitching?	None, each stay modeled independently	None, each admission modeled independently	None, patienthealthsystemstayid never used	None; structurally not possible (no patient-level key released)
Notes. (i) All four vasopressor rates target mcg/kg/min (weight-normalized): eICU normalizes explicitly, Amsterdam converts via the dose-unit “gamma” rule divided by patient weight, MIMIC uses its native mimic_derived.sofa units, and the HiRID values are passed through from an upstream extract. (ii) meanbp_min is mmHg everywhere, but Amsterdam stores the hourly maximum of mean arterial pressure while eICU stores the hourly minimum—a minor cross-database aggregation difference that local retraining absorbs. (iii) A single hourly PaO_2_/FiO_2_ ratio is routed to the ventilated or non-ventilated column by ventilation status; the other column is set to 0. (iv) No database applies a sedation/intubation correction to GCS. (v) Constant-imputed cells (used only by the direct-transfer condition; local retraining re-learns from the variables that carry information): Amsterdam Charlson (missing/uninformative); HiRID dopamine rate (0), Charlson (5.78, the MIMIC training mean), and the three admission-type columns (Medical = 1, the two surgical columns = 0). (vi) MIMIC-IV’s icu_expire_flag is a date-only proxy (death on or before the calendar day of ICU outtime), coarser than the exact discharge-status fields used by the other three databases, because MIMIC-IV’s de-identified date of death carries no time-of-day. (vii) AmsterdamUMCdb’s hospital_expire_flag is copied directly from icu_expire_flag rather than computed independently, so the two are identical by construction for this database; unlike MIMIC-IV and eICU-CRD, Amsterdam cannot distinguish an in-ICU death from a later in-hospital, post-ICU death, and any hospital-level mortality figure reported for Amsterdam elsewhere in this paper should be read as identical to its ICU-level figure. (viii) In HiRID, 164 of 9150 stays (1.8%) have a missing (NaN) discharge_status and are mapped to Exitus = 0, that is, treated as survivors, a data-quality issue not present in the other three databases. (ix) In all four databases, the discharge outcome is defined and modeled per ICU stay, not per patient; none of the four ETL pipelines links the repeated ICU stays of the same individual.				

## Appendix B. Missingness by Variable and Database

Table A1 reports, for each of the 16 harmonized input variables, the percentage of hourly rows that still hold no value once the two per-variable rules described in Section 2.3, the zero-fill and the LOCF, have been applied, and before the first-hour median fill, together with the rule that applies to each variable (zero-fill, LOCF, or none in the case of the static fields). The four databases are measured at the same point, so the columns are directly comparable. For the six zero-filled variables, the figure is 0.0% everywhere by construction, since that rule leaves no gap behind. For the five variables carried forward, it is the share of hours at the beginning of a stay that precede the first measurement of that variable, which is what the first-hour median fill has to resolve; a laboratory value measured once a day counts as present for every later hour of the stay, so these percentages describe how late in the stay a variable becomes available, and not how often it is measured.

**Table A1:** Percentage of hourly rows with no value available, by variable and database, after the zero-fill and the LOCF rules of Section 2.3 have been applied once and before the first-hour median fill. The label [constant-imputed] marks a field that takes a single fixed value for every stay in that database (see Appendix A).

Variable	Imputation Rule	MIMIC-IV	AmsterdamUMCdb	eICU-CRD	HiRID
pao2fio2ratio_novent	zero-fill	0.0%	0.0%	0.0%	0.0%
pao2fio2ratio_vent	zero-fill	0.0%	0.0%	0.0%	0.0%
gcs_min	LOCF	1.1%	19.7%	4.8%	0.7%
rate_epinephrine	zero-fill	0.0%	0.0%	0.0%	0.0%
rate_norepinephrine	zero-fill	0.0%	0.0%	0.0%	0.0%
rate_dopamine	zero-fill	0.0%	0.0%	0.0%	0.0% [constant-imputed]
rate_dobutamine	zero-fill	0.0%	0.0%	0.0%	0.0%
meanbp_min	LOCF	0.2%	0.8%	2.4%	0.1%
bilirubin_max	LOCF	42.9%	17.8%	48.0%	37.9%
platelet_min	LOCF	6.3%	2.6%	17.0%	6.4%
creatinine_max	LOCF	5.7%	0.6%	14.4%	9.5%
admission_age	none	0.0%	0.0%	<0.1%	0.0%
charlson_comorbidity_index	none	0.0%	0.0% [constant-imputed]	0.0%	0.0% [constant-imputed]
admission_type_Medical	none	0.0%	0.0%	0.0%	0.0% [constant-imputed]
admission_type_ScheduledSurgical	none	0.0%	0.0%	0.0%	0.0% [constant-imputed]
admission_type_UnscheduledSurgical	none	0.0%	0.0%	0.0%	0.0% [constant-imputed]

## Appendix C. Whole-Stay Versus Terminal-Window Mortality Discrimination

The mortality results in the main text (Table 2) are measured on the final 48-h window of each stay, so they describe how well the model separates patients who are already close to the outcome. For completeness, we also measured mortality discrimination across the whole stay, applying the mortality model at every 48-h window from the first complete window onwards (many windows per stay) and comparing the predictions against each stay’s eventual ICU mortality. As expected, whole-stay discrimination is markedly lower than terminal-window discrimination on every database (Table A2), because early in a stay the physiology of the patients who will eventually die still resembles that of those who will survive. These values quantify the early-warning limitation described in the Discussion; they do not change the terminal-window results in Table 2, which are the intended operating point of the model. Two points should be kept in mind when reading Table A2. First, the whole-stay value is a new measurement rather than a figure reported in the first study, which also evaluated mortality on the final window of the stay. Second, the pooled whole-stay value is weighted by length of stay, so a database with longer stays (Amsterdam, with about 229 evaluation windows per stay against roughly 90 for the others) contributes many early, far-from-outcome windows and therefore has a lower pooled value that reflects its length-of-stay distribution as much as its model quality.

**Table A2:** Whole-stay versus terminal-window (final 48 h) mortality discrimination for the locally adapted (full) model on each database. The terminal-window values reproduce Table 2.

Database	Terminal-Window AUROC	Whole-Stay AUROC	Difference
MIMIC	0.965	0.777	−0.188
Amsterdam	0.974	0.699	−0.276
eICU	0.955	0.754	−0.201
HiRID	0.986	0.719	−0.267
